# Synthesis of π-conjugated network polymers based on triphenylamine (TPA) and tetraphenylethylene (TPE) as building blocks *via* direct Pd-catalyzed reactions and their application in CO_2_ capture and explosive detection†

**DOI:** 10.1039/c9ra02469g

**Published:** 2019-06-10

**Authors:** Lamaocao Ma, Hengchang Ma

**Affiliations:** Shaw Library, Northwest Normal University Lanzhou Gansu 730070 P. R. China; Key Laboratory of Eco-Environment-Related Polymer Materials Ministry of Education, Key Laboratory of Polymer Materials Ministry of Gansu Province, College of Chemistry and Chemical Engineering, Northwest Normal University Lanzhou Gansu 730070 P. R. China mahczju@hotmail.com

## Abstract

In this study, we report the synthesis of π-conjugated network polymers *via* palladium-catalyzed direct arylation polycondensation of triphenylamine (TPA) and tetraphenylethylene (TPE) with different active substrates. Moreover, six conjugated porous polymers were obtained (named as TPA-TPA-MA, TPA-PB-MA, TPA-TFB-MA, TPA-TPE-MA, TPE-PB-MA, and TPE-TFB-MA). Then, the fluorescence properties in the solid and dispersed states, the corresponding microporous structures, and the Brunauer–Emmett–Teller (BET) surface areas of all polymers were well studied. Among the obtained materials, TPA-PB-MA possessed not only largest BET surface area (686 m^2^ g^−1^) and largest pore volume (0.716 cm^3^ g^−1^), but also the smallest pore size of 0.823 nm. These properties are very beneficial for the application of TPA-PB-MA in CO_2_ storage and PA sensing. At 1 bar, TPA-PB-MA demonstrated the significant CO_2_ uptake of 2.70 and 1.35 mmol g^−1^ at 273 and 298 K, respectively. Furthermore, TPA-PB-MA was most sensitive and selective towards PA recognition. The *K*_SV_ constant was measured as 4.0 × 10^4^ M^−1^.

## Abstract 2

In this study, we report the synthesis of π-conjugated network polymers *via* palladium-catalyzed direct arylation polycondensation of triphenylamine (TPA) and tetraphenylethylene (TPE) with different active substrates.

## Graphical abstract

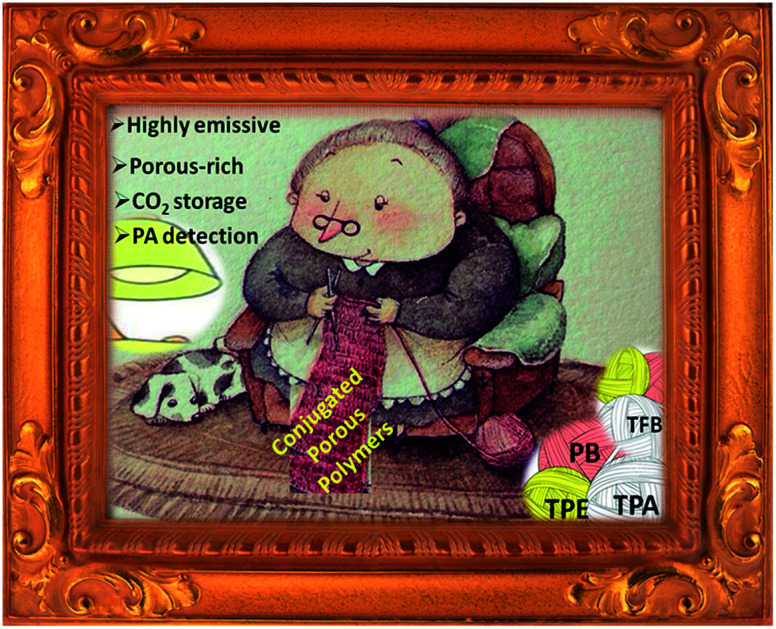

## Introduction

Conjugated polymers with a rigid framework are fascinating materials due to their extensive π-conjugation and the corresponding light-emitting behaviors.^1–3^ Furthermore, conjugated porous polymers are more attractive because of their large surface areas, large capacity for gas storage, and interesting optical property for stimulus sensing;^4–13^ certainly, the concept of aggregation-induced emission (AIE) has established a colorful emission platform for the optical science study.^14–17^ In recent years, Tang's group has found that when AIEgens are incorporated into polymer networks, the polymer networks become able to partially interlock the free rotation of the embedded AIEgens.^18,19^ As a result, molecular rotations can be restrained to some extent; this leads to more emissive polymers not only in the powder but also in the solution state.^20,21^ Therefore, polymerization-promoted AIE systems have been designed and developed in recent years.^22–25^

By integrating the desirable AIE feature with conjugated polymer characteristics, attractive materials could be generated.^26,27^ Note that in conjugated polymer systems, the fluorescent cores have multiple exciton transfer channels, which can lead to high sensitivity in the sensing processes by the expression of fluorescence signals.^28,29^ Due to the advantages of conjugated polymers, it is believed that the exploration of AIE-active conjugated polymers with their potential applications will be a hot research topic in the coming years.^30–33^

In our previous studies, a series of AIE-active polymers were obtained, which demonstrated tunable fluorescence properties and sensing performances towards an external stimulus.^34^ However, a systematic study of conjugated polymers and their real applications still remain to be fully exploited. Inspired by this research point, in the present study, we synthesized several AIE-active conjugated porous polymers by reliable synthetic methods from commercially available starting materials. In detail, TPA-TPA-MA, TPA-PB-MA, and TPA-TFB-MA were first synthesized using the fluorescent component triphenylamine (TPA). After polymerization, small functional tails of methyl acrylate were introduced into the organic frameworks by the Heck cross-coupling reaction. Moreover, since tetraphenylethylene (TPE) is the most typical AIE-active compound, polymerization can promote the TPE derivatives to be more emissive in rigid networks.^35–37^ Then, the TPE-based polymers TPA-TPE-MA, TPE-PB-MA, and TPE-TFB-MA were obtained. Among them, TPE-PB-MA presented the highest fluorescence quantum yield of 4.18% and the maximum emission wavelength of 544 nm. In further applications, we expect that due to the presence of the electron-donor TPA and TPE cores, all conjugated polymers would be employed as fluorescent platforms for the detection of electron-deficient explosives, especially for the detection of picronitric acid (PA).^38,39^ In another way, all polymers with a three-dimensional porous architecture would possibly allow gas sorption and adsorption;^40–42^ for this purpose, this study was focused on the investigations of conjugated polymers with different physical properties, and all results led us to make a clear understanding of the primary relationships between their chemical structures, photophysical properties and adsorption behaviors. Finally, we explored their promising applications in gas storage and specific explosive sensing. Therefore, our findings would be very useful to the design and synthesis of fluorescent porous polymers and also open the avenue to produce advanced polymers with versatile applications in future studies.

## Experimental

### Materials

Tris(4-iodophenyl)amine (TPA-3I, 98%), 4,4′-dibromobenzophenone (98%), benzene-1,4-diboronic acid (PB, 97%), 9,9-dioctylfluorene-2,7-bis(boronic acid pinacol ester) (TFB, 98%), tetrakis(triphenylphosphine)palladium(0) (Pb(PPh_3_)_4_ 98%), potassium carbonate (K_2_CO_3_, 99%), methyl acrylate (MA, 99%), palladium acetate (Pd(OAc)_2_ 99%), 1,3-bis(diphenylphosphino)propane (DPPP, 98%), triethylamine (Et_3_N 99%), zinc, titanium tetrachloride (TiCl_4_, 99%), nitrobenzene (NB, 98%), *p*-nitrotoluene (*p*-NT, 98%), *p*-nitrobenzaldehyde (*p*-NBD, 98%), *p*-chloronitrobenzene (*p*-CNB, 98%), *m*-dinitrobenzene (*m*-BD, 98%) and picric acid (PA, 98%) were purchased from Aladdin Co. Nitrogen with the purity of 99.99% was obtained from a commercial source. Other reagents, such as *N*,*N*-dimethylformamide (DMF), acetone, methanol, tetrahydrofuran (THF), dichloromethane (DCM), chloroform and HCl were of analytical grade and purchased from Energy Chemical Company.

^1^H NMR (600 MHz) and ^13^C NMR (150 MHz) spectra were obtained using the MERCURY spectrometer at 25 °C, and all the NMR spectra were referenced to the solvent. Solid-state NMR experiments were performed using the JNM-ECZ600R 600 MHZ NMR spectrometer. The ^13^C cross-polarization magic angle spinning (CP/MAS) spectra were acquired using a 4 mm double-resonance MAS probe at the MAS rate of 10.0 kHz with the contact time of 2 ms (ramp 100) and the pulse delay of 3 s. UV-visible absorption spectra (UV) were obtained using the TU-1901 spectrometer. Fluorescence spectra and fluorescence quantum yields were measured using the Fluoro Sens 9003 fluorescence spectrophotometer. All the samples were prepared according to the standard methods. Fourier transform infrared (FTIR) spectra were obtained *via* the DIGIL FTS3000 spectrophotometer using KBr tablets. The TG analysis of the adsorbent was performed using Germany Netzach STA449C at the heating rate of 10 °C min^−1^ under a nitrogen atmosphere. Scanning electron microscopy (SEM) was carried out using ULTRA plus (Germany, Carl Zeiss) at 5 kV. The powders of the polymers were examined by an X-ray diffractometer (Rigaku, D/Max-2400). The samples were characterized in terms of specific surface area, pore volume, and pore diameter using the adsorption of nitrogen (America, Autosorb-iQ2-MP) at 77 K. The BET surface area was determined from the isotherms using the Brunauer–Emmett–Teller equation (BET). The pore size distribution was calculated from the adsorption branch using the nonlocal density functional theory (NLDFT). Gas uptake experiments were performed using Autosorb-iQ2-MP (Quantachrome Instruments). All the gases used were of high purity.

### Synthesis of TPA-TPA-MA

The synthesis of TPA-TPA was performed by Ullmann polymerization. Tris(4-iodophenyl)amine (TPA-3I, 3.115 g, 5 mmol) and Pd(OAc)_2_ (0.056 g, 0.15 mmol) were added to a solution of K_2_CO_3_ (2.073 g, 15 mmol) and DPPP (0.206 g, 0.5 mmol) in dehydrated DMF (150 mL) under a N_2_ atmosphere, and the mixture was heated at 110 °C for 72 h to afford a brown suspension. After cooling down to ambient temperature, concentrated HCl (10 mL) was added to the mixture. After filtration, the residue was washed with H_2_O, MeOH, CHCl_3_, THF, and acetone, extracted by Soxhlet with H_2_O, methanol, CHCl_3_, acetone, and THF for 1 day, and dried at 100 °C under vacuum for 24 h to afford yellow powder (2.1 g, 67% yield). TPA-TPA (1.8 g), MA (0.774 g, 9 mmol), Pd(OAc)_2_ (0.1 g, 0.045 mmol), DPPP (0.37 g, 0.9 mmol) and Et_3_N (2.24 g, 10 mmol) were added to 100 mL of dry DMF. Then, the reaction mixture was stirred for 72 h at 100 °C under a N_2_ atmosphere. After cooling down the reaction mixture to ambient temperature, the residue was isolated by filtration, washed with H_2_O, MeOH, CHCl_3_, THF, and acetone, and dried in vacuum to obtain a bright yellow solid (0.9 g, 65% yield). IR (KBr): 3031, 1703, 1601, 1485, 1435, 1324, 1267, 1177, 1103, 820, 740, 690, 598, and 529 cm^−1^.

### Synthesis of TPA-PB-MA

The synthesis of TPA-PB was performed by Suzuki polymerization. A dried 250 mL round-bottom flask was charged with TPA-3I (1.869 g, 3 mmol), PB (0.747 g, 4.5 mmol), K_2_CO_3_ (1.242 g, 9 mmol) and Pb(PPh_3_)_4_ (0.3 mmol, 0.346 g). Then, the mixture of THF (80 mL) and MeOH (80 mL) was added, and the reaction was refluxed at 120 °C for 48 h under a N_2_ atmosphere. The mixture was allowed to cool at ambient temperature and poured into water. The precipitate was obtained by filtration, thoroughly washed with H_2_O, MeOH, CHCl_3_, THF, and acetone, extracted by Soxhlet with H_2_O, methanol, CHCl_3_, acetone, and THF for 1 day, and dried at 100 °C under vacuum for 24 h to afford a white solid (1.335 g, 78% yield). TPA-PB (0.9 g), MA (0.517 g, 6 mmol), Pd(OAc)_2_ (0.05 g, 0.025 mmol), DPPP (0.186 g, 0.45 mmol) and Et_3_N (1.347 g, 6 mmol) were added to 80 mL of dry DMF using procedures similar to those used for TPA-TPA-MA. A gray solid was obtained in the yield of 68%. IR (KBr): 3026, 1599, 1477, 1326, 1282, 1183, 1116, 995, 954, 813, 743, 673, 592, and 522 cm^−1^.

### Synthesis of TPA-TFB-MA

The synthesis of TPA-TFB was conducted by Suzuki polymerization. A dried 250 mL round-bottom flask was charged with TPA-3I (1.246 g, 2 mmol), TFB (1.927 g, 3 mmol), K_2_CO_3_ (1.242 g, 9 mmol) and Pb(PPh_3_)_4_ (0.346 g, 0.3 mmol). Then, the mixture of THF (80 mL) and MeOH (80 mL) was added. Using procedures similar to those used for TPA-PB, a yellowish brown solid was obtained in an 80% yield. TPA-TFB (0.89 g), MA (0.517 g, 6 mmol), Pd(OAc)_2_ (0.05 g, 0.025 mmol), DPPP (0.186 g, 0.45 mmol) and Et_3_N (1.347 g, 6 mmol) were added to 80 mL of dry DMF using procedures similar to those used for TPA-TPA-MA. A khaki solid was obtained in a 65% yield. IR (KBr): 2930, 2851, 1605, 1509, 1463, 1311, 1280, 1181, 1108, 1012, 956, 888, 809, 735, 623, and 538 cm^−1^.

## Results and discussion

### Polymer synthetic methods

**Scheme 1:**
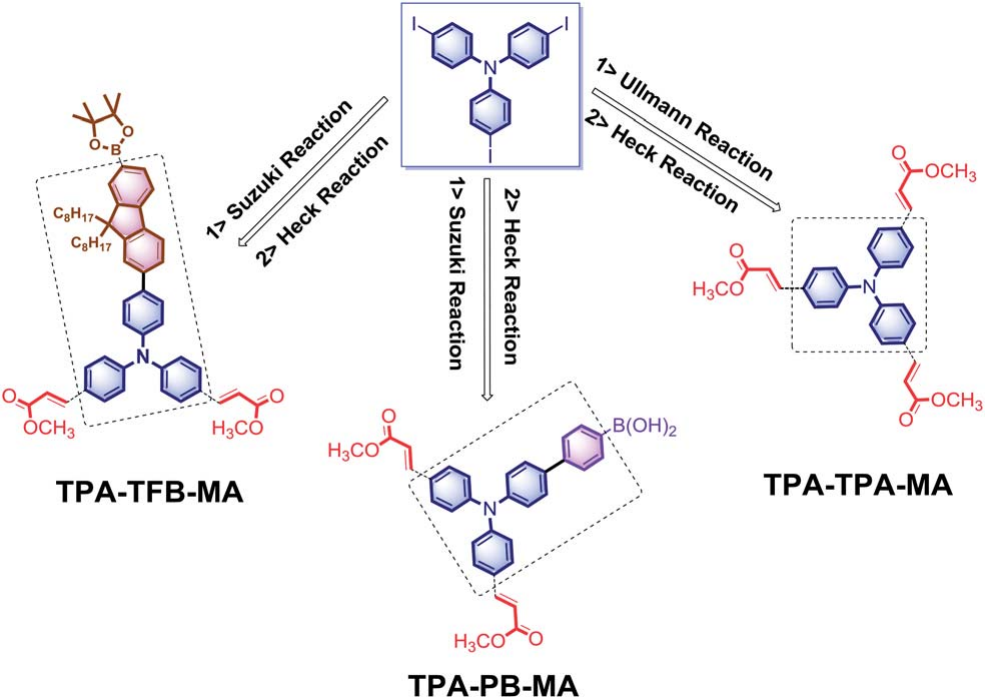
The synthesis of TPA-TPA-MA, TPA-PB-MA, TPA-TFB-MA.

Initially, the electron-rich and totally active TPA-3I was employed as the starting material. Due to the presence of C–I bonds, the following reactions could be trustingly utilized to prepare the conjugated networks. The Ullmann reaction between TPA-3I was carried out, producing the π-conjugated polymers named as TPA-TPA. Then, the electron-deficient tails of methyl acrylate (MA) fully terminated the C–I bonds by the Heck cross-coupling reaction; this led to the formation of TPA-TPA-MA. Then, the cross-coupling of TPA-3I and PB by the Suzuki reaction was performed, and MA was attached; this formed the desirable polymer TPA-PB-MA. It was reasonably expected that the long alkyl chains were able to promote the solubility of the polymers in organic solvents and the processability of macromolecules. Then, TFB was employed to couple with the TPA cores, and TPA-TFB was produced. Finally, MA was attached to TPA-TFB; this resulted in TPA-TFB-MA. Note that the polymers TPA-TPA, TPA-PB, and TPA-TFB were very conveniently obtained from the reaction media by simple filtration. After washing several times with hot THF, sponge-like powders were produced; at first, all of them were less emissive but porous in the solid phase. Finally, to enhance their emission performance and processability in organic solvents and also their chemical stability due to the presence of atomic iodine, MA was fully terminated by the C–I bonds *via* the Heck cross-coupling reaction, leading to the corresponding polymers TPA-TPA-MA, TPA-PB-MA, and TPA-TFB-MA (Scheme 1). Obviously, the MA-modified materials exhibited stronger emission performances than the precursors TPA-TPA, TPA-PB, and TPA-TFB. This phenomenon was due to the formation of electron-donating and -accepting systems. Moreover, nitrogen-rich conjugated porous polymers are of significant interest.^43–45^ Therefore, the TPA-based polymers TPA-TPA-MA, TPA-PB-MA, and TPA-TFB-MA were expected to have broad applications in different research fields in the future.

**Scheme 2:**
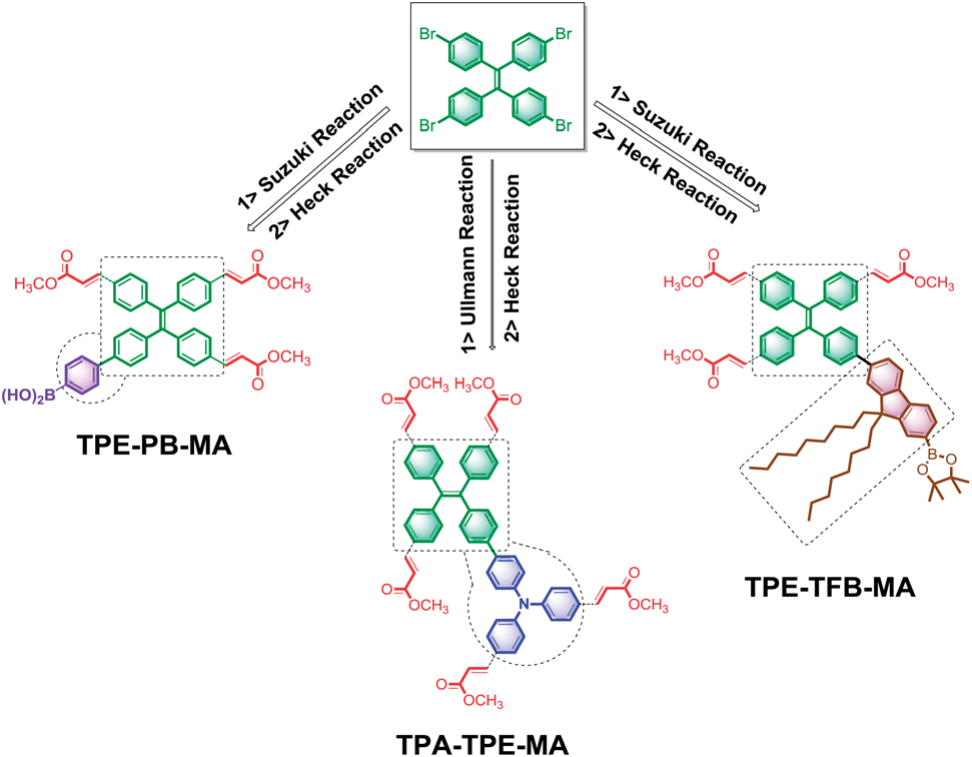
The synthesis of TPA-TPE-MA, TPE-PB-MA, and TPE-TFB-MA.

TPE has been disclosed as a typical AIEgen by Tang's group.^46^ When TPE is incorporated into polymer networks, the TPE derivatives become more emissive as abovementioned. Therefore, in the following studies, we used TPE-4Br as the starting material, and several compounds were identified as the reaction substrates. TPA is a highly electron-rich ACQ molecule. The cross-coupling of TPA with the typical AIEgen TPE by the Ullmann reaction could be expected to produce emissive framework materials. Then, TPA-TPE was obtained. Moreover, boric acid compounds are less toxic and more stable under normal storage conditions, which can be used for versatile chemical bond formation.^47^ Thus, the cross-coupling of TPE-4Br and PB by the reliable Suzuki reaction was performed, leading to the TPE-PB polymers. Accordingly, due to the presence of soft long alkyl chains, TFB was used to attach to the TPE cores *via* the Suzuki reaction, and the polymer TPA-TFB was formed. Herein, we noted that because of the typical AIE property, the TPE-based polymers TPA-TPE, TPE-PB, and TPA-TFB demonstrated a highly yellow emissive color even without decoration with MA. However, to improve the processability and chemical stability, MA was attached *via* the Heck cross-coupling reaction to fully terminate the C–Br bonds; this solid-state synthetic method led to the corresponding polymers TPA-TPE-MA, TPE-PB-MA, and TPE-TFB-MA (Scheme 2).

### Polymer structure characterization

**Fig. 1:**
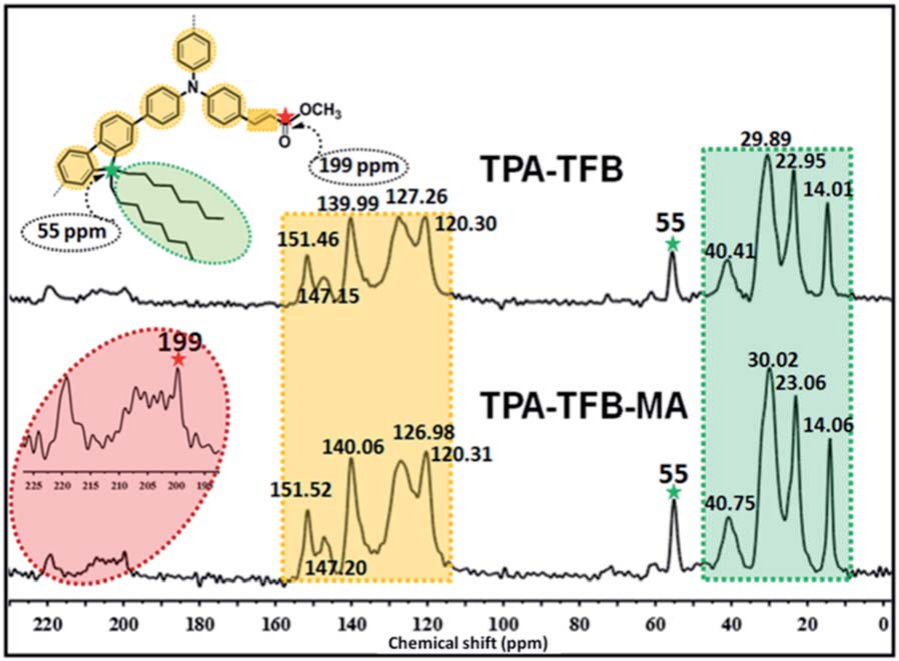
Solid-state ^13^C CP/MAS NMR spectra of TPA-TFB (top) and TPA-TFB-MA (bottom) obtained at the MAS rate of 10 kHz and the contact time of 2 ms.

**Fig. 2:**
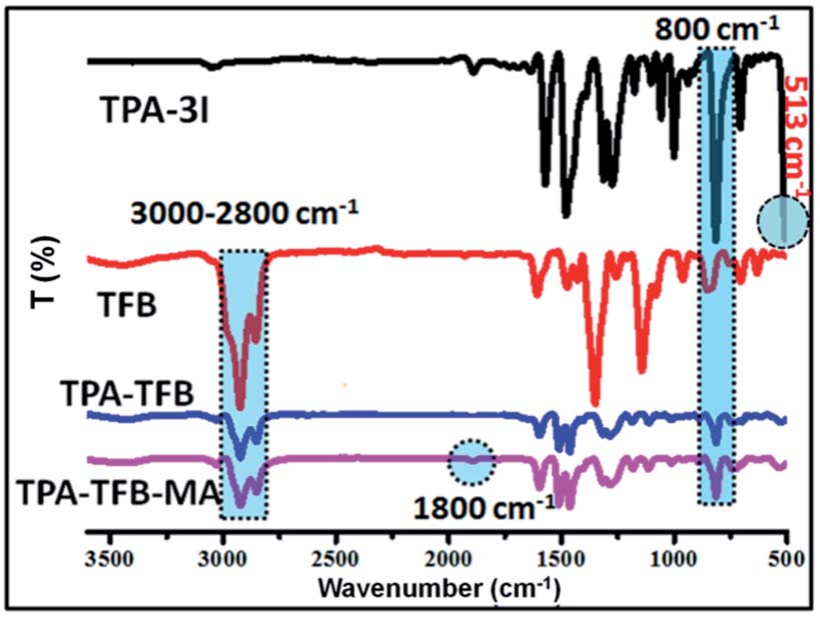
FTIR spectra of TPA-3I, TFB, TPA-TFB and TPA-TFB-MA.

To confirm the predicted structures of conjugated polymers, we chose TPA-TFB and TPA-TFB-MA as typical samples to verify their structures by solid-state ^13^C cross-polarization magic-angle spinning (CP/MAS) NMR spectra. The signal assignment of carbons in the polymer skeletons is depicted in Fig. 1. In detail, the signals in the range from 14 to 40 ppm indicated the presence of sp^3^-hybridized carbons from TFB for both TPA-TFB and TPA-TFB-MA. The isolated peak at 55 ppm was ascribed to the signals of a quaternary carbon atom from TFB (green star). In addition, the peaks for the unsaturated carbons from all phenyl rings were located from 120 to 150 ppm for TPA-TFB and TPA-TFB-MA. Moreover, the characteristic peak of the corresponding C=O carbon appeared at 199 ppm for TPA-TFB-MA (red star). To further support the successful formation of these polymers, Fourier-transform infrared (FTIR) spectroscopy was applied (Fig. 2 and S1–S5†). Accordingly, the formation of TPA-TFB-MA was taken as an example. The spectra clearly showed the appearance of the C–I bonds for TPA-3I only (513 cm^−1^) and the disappearance of the C–I bonds for TPA-TFB and TPA-TFB-MA. Moreover, the intensified bands appearing at around 3000–2800 cm^−1^ were assigned to the stretching vibrations of the C–H bonds from the saturated hydrocarbon chains of TFB. These peaks confirmed the attachment of TFB to the polymers with high efficiency by Suzuki polymerization. Finally, the small peak at 1800 cm^−1^ also verified the successful coupling of TPA-TFB with MA. The broad peaks obtained in the powder X-ray diffraction (PXRD) patterns of these polymers revealed that all polymers were amorphous (Fig. S6–S11†). The field emission scanning electron microscopy (FE-SEM) images demonstrated irregular sub-micrometer morphology (Fig. S12†). The high polymerization degree of these conjugated polymers could also be supported by their excellent thermal stability. As demonstrated in Fig. S13,† all the conjugated polymers were significantly stable under a nitrogen atmosphere even at 500 °C.

### Fluorescence property of polymers in the solid state and dispersed state

**Fig. 3:**
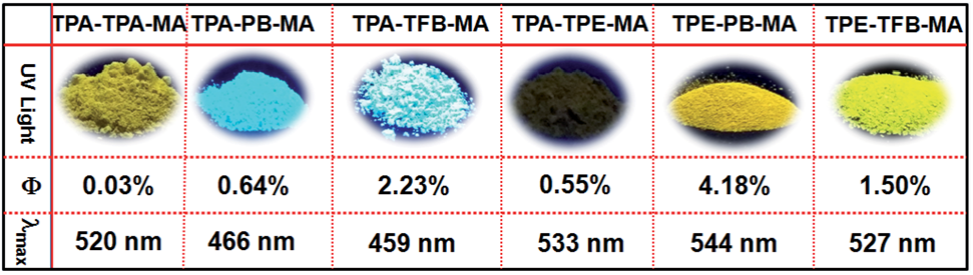
The corresponding images under UV light illumination at 365 nm, fluorescence quantum yields and the maximum emission wavelength.

Obviously, compared to the monomers used herein, which are almost non emissive or less emissive, the corresponding conjugated polymers exhibit highly emissive performances from blue to yellow colors in the solid state (Fig. 3 and S15†). Among them, TPE-PB-MA presented the longest emission wavelength of 533 nm and the highest fluorescence quantum yield of 4.18% upon excitation at 399 nm. In addition, TPA-PB-MA displayed the moderate emission wavelength of 459 nm and the fluorescence quantum yield of 2.23%. According to our considerations, these interesting fluorescence behaviours were partially dependent on the substrates of 1,4-phenylenebisboronic acid, which was used as one monomer for the synthesis of TPE-PB-MA and TPA-PB-MA. In contrast, TPA-PB-MA and TPA-TFB-MA emitted a strong blue color with the maximum emission wavelength below 500 nm. The others emitted yellow colour with the maximum emission wavelengths more than 500 nm.

**Fig. 4:**
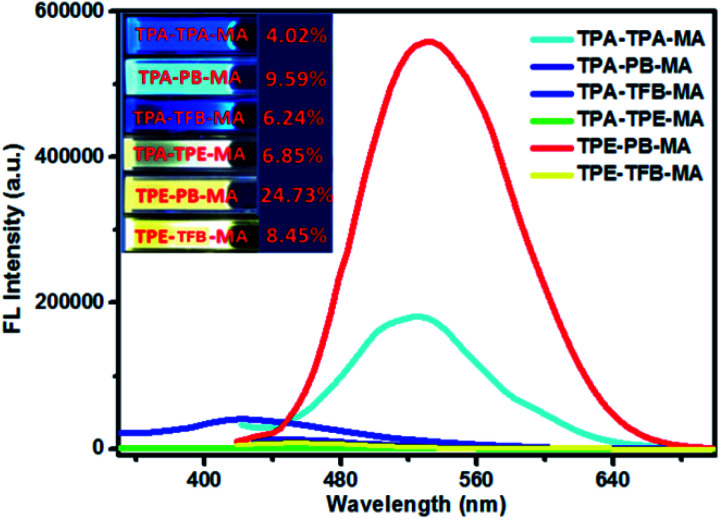
Fluorescence spectra of the polymers dispersed in THF and their fluorescence quantum yields. The corresponding images were obtained under UV light illumination at 365 nm. (The excitation wavelength of TPA-TPA-MA, TPA-TPE-MA and TPA-TFB-MA was 325 nm; the excitation wavelength of TPA-PB-MA, TPE-PB-MA and TPE-TFB-MA was 399 nm.)

We also investigated the emission behaviours of all polymers in the solution state (Fig. 4). Due to their very good dispersion ability in THF, these polymers could form stable solutions in THF even after storage at room temperature for months. In sharp contrast, all polymers displayed enhanced fluorescence quantum yields as compared to those in the solid state. Especially, TPE-PB-MA and TPA-PB-MA presented the highest fluorescence quantum yields of 24.73% and 9.59%, respectively, upon excitation at 399 nm. This dispersion ability in THF was possibly ascribed to their porous nature and the short tails of MA.

### Gas-adsorption properties

**Fig. 5:**
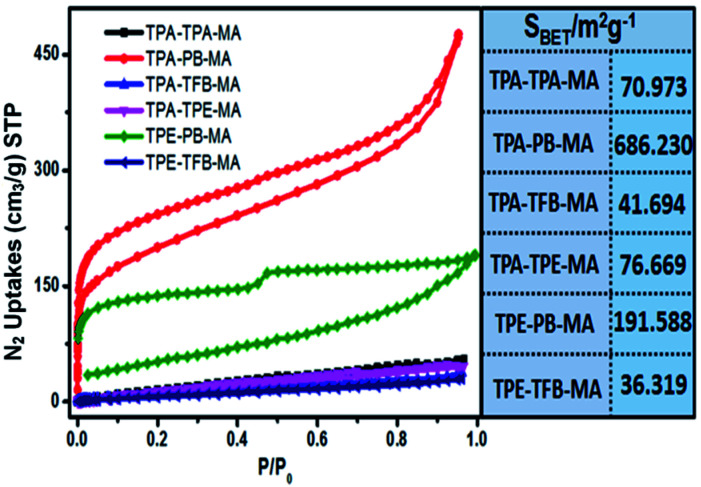
N_2_ adsorption/desorption isotherms of conjugated polymers and the corresponding BET surface areas.

Then, sorption isotherms for N_2_ at 77 K were measured and showed a type I and IV isotherm. The Brunauer–Emmett–Teller (BET) surface areas were estimated and are illustrated in Fig. 5. Obviously, TPA-PB-MA and TPE-PB-MA presented the largest surface area of 686 and 191 m^2^ g^−1^, respectively. Correspondingly, the pore volumes were calculated as 0.716 and 0.278 cm^3^ g^−1^ for TPA-PB-MA and TPE-PB-MA, respectively. Moreover, we found that the long alkyl chain-modified polymers TPA-TFB-MA and TPE-TFB-MA demonstrated smallest BET surface areas (41.6 and 36.3 m^2^ g^−1^) and pore volumes (0.055 and 0.043 cm^3^ g^−1^), respectively. This result exhibited the relationship between their chemical structures and corresponding porosity, where the long alkyl chains were probably less beneficial to the formation of the polyporous materials. The pore size distribution was determined by fitting the uptake branch of the N_2_ isotherm using the nonlocal-density-functional-theory (NLDFT) method. In Fig. S16 and Table S1,† we can see that the pore size distributions of all polymers are in the range from 0.823 to 5.888 nm. Moreover, we found that TPA-PB-MA possessed not only largest BET surface area (686 m^2^ g^−1^) and pore volume (0.716 cm^3^ g^−1^), but also the smallest pore size of 0.823 nm. This property is very beneficial for the application of TPA-PB-MA in gas storage and sensing. This result also demonstrated that 1,4-phenylenebisboronic acid has the potential to be used as a building block for the formation of conjugated porous polymers.

**Fig. 6:**
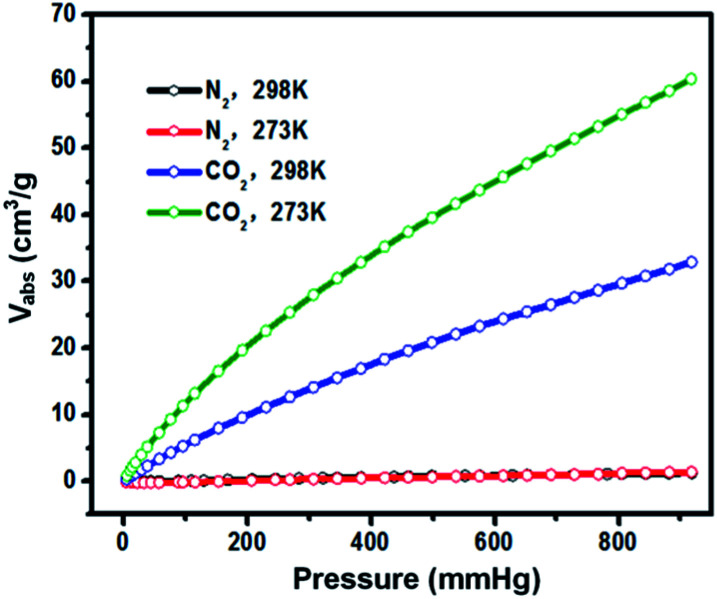
Adsorption isotherms of CO_2_ and N_2_ for TPA-PB-MA at 298 and 273 K.

**Fig. 7:**
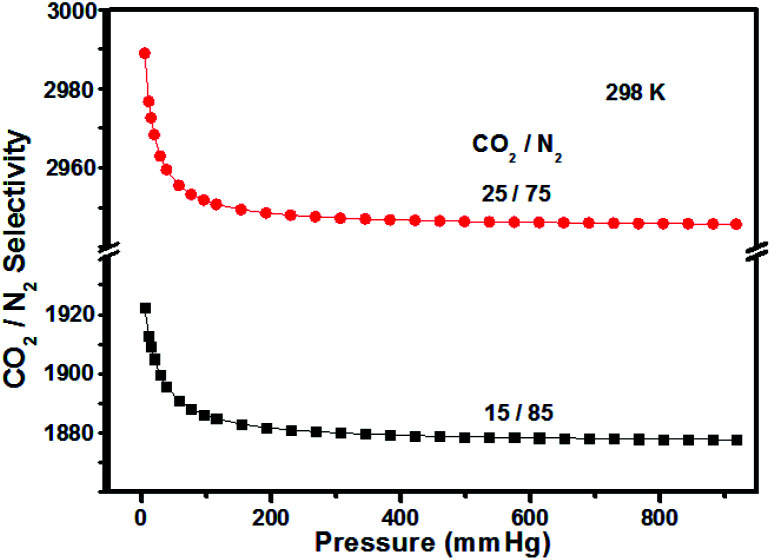
CO_2_ selectivity of TPA-PB-MA over N_2_ using 15/85 and 25/75 compositions of CO_2_/N_2_ gas phases at 298 K for calculations.

In the following research steps, due to the electron-rich structure and porosity of TPA-PB-MA, we investigated the CO_2_ and N_2_ uptake capacities of TPA-PB-MA under different conditions. The CO_2_ adsorption isotherms of TPA-PB-MA were measured at 273 and 298 K (Fig. 6). At 1 bar, TPA-PB-MA demonstrated the significant CO_2_ uptake of 2.70 mmol g^−1^ at 273 K and 1.35 mmol g^−1^ at 298 K. These values are higher than those reported for most of the previously synthesized porous materials such as polyaniline@MIL-101 (<2.26 mmol g^−1^),^48^ covalent organic frameworks (COF-1, 5, 8, 10, 102, 103 and TpBa, 1.38–2.37 mmol g^−1^),^49^ and CMPs (CMP-0, 5, TCMP-5, TFM-1, CMP-1-NH_2_, and CMP-1-COOH, 1.1–2.1 mmol g^−1^).^50–52^ The N_2_ uptake capacity of TPA-PB-MA was 0.062 mmol g^−1^ at 273 K and 0.049 mmol g^−1^ at 298 K. Therefore, these results clearly verified that the TPA-PB-MA network could be a promising candidate as an adsorbent for CO_2_ gas. Moreover, the isosteric heat of adsorption (*Q*_st_) was calculated based on the Clausius–Clapeyron equation using single gas adsorption isotherms. The initial *Q*_st_ values for CO_2_ adsorption were found to be as high as 12.0 kJ mol^−1^ for TPA-PB-MA. This result evidenced a very favorable physical interaction between the adsorbed CO_2_ molecules and the porous surfaces. Moreover, the CO_2_ uptake capacity and high CO_2_ selectivity over N_2_ are critical factors in the future real application. Therefore, the ideal adsorption solution theory (IAST) model (Fig. S18, see details in the ESI†) was also applied to predict the separation selectivity of CO_2_/N_2_ gas mixtures (15/85 and 25/75) on TPA-PB-MA in the entire pressure region at 298 K (Fig. 7). These results demonstrated that there was no radical decrease in the selectivity with an increase in pressure, and TPA-PB-MA presented dominant CO_2_-selectivities over N_2_ and the potential ability to separate gases.^53,54^

### PA sensing performances

**Fig. 8:**
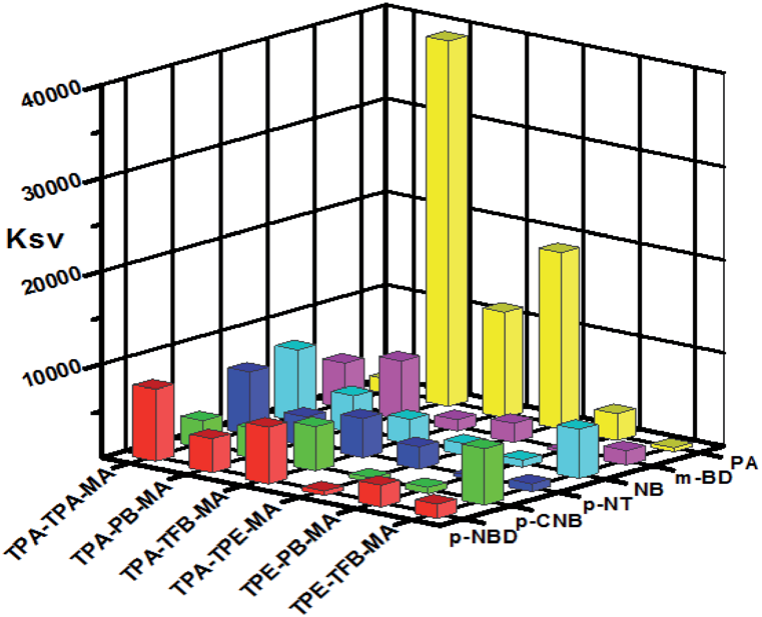
Fluorescence quenching efficiencies of polymers for different analytes. The *z*-axis denotes the Stern–Volmer constant *K*_sv_.

**Fig. 9:**
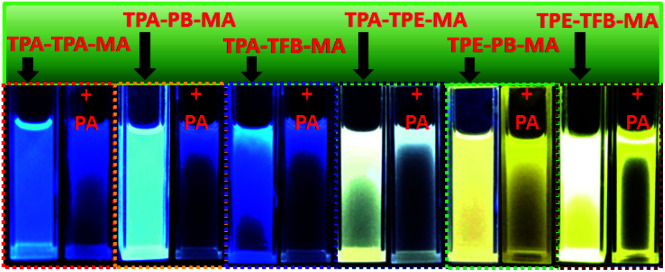
The corresponding fluorescence images of conjugated polymers before and after the addition of PA in THF obtained under UV irradiation at 365 nm.

**Fig. 10:**
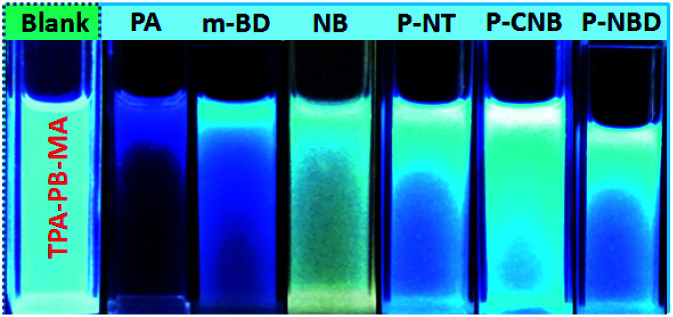
The corresponding fluorescence images of TPA-PB-MA before and after the addition of the analytes in THF obtained under UV irradiation at 365 nm.

As abovementioned, our conjugated networks mainly consisted of the fluorescence-active triphenylamine (TPA) and tetraphenylethylene (TPE), known as the electron-rich compounds. Picric acid (PA), as an electron deficient molecule, has emerged as a potential explosive due to its high energy release power. Thus, considering the social life safety, effective monitoring and detection of PA in trace amounts is really important.^55,56^ By gradually adding the analytes of PA and other nitroarenes, the fluorescence intensity of the polymer solutions decreased to some extent. Fig. 8 demonstrates the *K*_SV_ constants measured by the Stern–Volmer equation for the fluorescence quenching of six polymers with different nitroarenes. We can see that the TPA-consisted polymers TPA-PB-MA, TPA-TFB-MA, and TPA-TPE-MA are particularly sensitive towards PA sensing. Especially, TPA-PB-MA was most sensitive and selective towards PA recognition (Fig. 9 and 10). The *K*_SV_ constant was measured as 4.0 × 10^4^ M^−1^, which was compared with that of other polymer chemosensors reported in the literature (Table S3†). We have previously postulated that FRET is the major quenching mechanism, which is dependent on the spectral overlap of a FRET donor, *i.e.*, TPA-PB-MA, with the UV-vis spectrum of PA (Fig. S26†).^57^ In addition to this, due to its largest BET surface area, largest pore volume, and smallest pore size, TPA-PB-MA displayed most sensitive and selective sensing performance towards PA.

## Conclusions

Although it is really challenging to investigate conjugated porous polymers, their meaningful and extraordinary applications in different research fields motivate researchers for their further investigation. This study improved our understanding of the primary relationships between chemical structures, photo-physical properties, adsorption behaviors, *etc.* of these polymers. *Via* our studies, we could conclude that 1,4-phenylenebisboronic acid (PB) could be used as a promising building block for the formation of conjugated porous polymers. Moreover, long alkyl chains are probably less beneficial to the formation of porous materials. Correspondingly, TPA-PB-MA exhibited highest adsorption capacity for CO_2_ and best sensing ability towards PA; this was probably ascribed to its largest BET surface area (686.230 m^2^ g^−1^), largest pore volume (0.716 cm^3^ g^−1^), and the smallest pore size of 0.823 nm. Thus, our findings are very important from two aspects: (i) the use of appropriate monomers is important for the design and synthesis of fluorescent porous polymers, especially from the fluorescence and porosity point of views, and (ii) the reliable synthetic procedures together with excellent pore structures and adsorption behaviors of TPA-PB-MA as compared to those of competitive materials highlight the potential applications of these conjugated porous polymers in gas storage and explosive detection.

## Conflicts of interest

There are no conflicts to declare.

## Supplementary Material

RA-009-C9RA02469G-s001
