# Supplementary material for: Synthesis of π-conjugated network polymers based on triphenylamine (TPA) and tetraphenylethylene (TPE) as building blocks via direct Pd-catalyzed reactions and their application in CO2 capture and explosive detection
Source: RSC Adv. 2019 Jun 10;9(32):18098–105. doi: 10.1039/c9ra02469g (PMC9064729; doi:10.1039/c9ra02469g)
Supplement: RA-009-C9RA02469G-s001 [file RA-009-C9RA02469G-s001.pdf]

Electronic Supplementary Material (ESI) for RSC Advances.
This journal is © The Royal Society of Chemistry 2019

Synthesis of π-Conjugated Network Polymers Based on Triphenylamine (TPA) and Tetraphenylethylene (TPE) as the Building Blocks *via* Direct Pd-Catalyzed Reactions and Their CO_2 Capture and Explosive Detection

Lamaocao Ma, Hengchang Ma

Table of Contents

Experimental Section......2

S1.1. Synthesis of 1,1,2,2-tetrakis(4-bromophenyl)ethane

S1.2-S1.7 Synthesis of conjugated polymers.

S1.8 Fluorescence quantum yield (Φ_F) measurement.

S1.9 Isosteric Heat of Adsorption (*Q*st) Calculations

S1.10 Ideal adsorption solution theory (IAST) calculations.

Characterization and Measurements......12

Fig. S1-S5 FT-IR data spectra......12

Fig. S6-S11 X-ray diffraction profiles......15

Fig. S12 FE-SEM images......18

Fig. S13 Thermal gravimetric analysis......19

Fig. S14 Absorption spectra studies in solid state......20

Fig. S15 Fluorescence spectra studies in solid state......21

Fig. S16 Pore size distribution......22

Table S1 Porosity parameter of conjugated polymers......23

Fig. S17 Adsorption and desorption isotherm of CO_2 and N_2......24

Fig. S18 Langmuir model fittings of N_2 and CO_2 isotherms......25

Scheme S1. Structures of the different tested nitroarenes......26

Table S2. The Ksv constants of conjugated polymers to the analytes......27

Table S3. Summary of K_{SV} of polymers......28

Fig. S19-S24 Fluorescence spectra of conjugated polymers to the analytes......29

Fig. S25 UV-Vis absorption spectra of PA and fluorescence spectra of TPA-PB-MA......35

References......36

S1.1 Synthesis of 1,1,2,2-tetrakis(4-bromophenyl)ethane (TPE-4Br).

3.4 g (10 mmol) of 4,4' Dibromobenzophenone and 2.62 g (40 mmol) of Zn powder was added to a 500 mL pre-dried three necked round-bottom flask with a magnetic stirrer and a water condenser. The flask was evacuated under vacuum and flushed with nitrogen for three times. 20 mL of THF was injected into the flask and the mixture was stirred under an ice-bath for 0.5 h. 1 mL (8 mmol) of $TiCl_4$ was injected drop-wise into the flask and the mixture was stirred again for 0.5 h on an ice bath. The mixture was refluxed at 70 °C overnight. After cooling and solvent evaporation, 100 ml of deionized water and 20 mL of potassium carbonate solution were added to the reaction mixture. Then 6M HCl was added to the reaction mixture to adjust the pH of the solution to be 7. The mixture was extracted with DCM and the organic layer was obtained. 1,1,2,2-tetrakis(4-bromophenyl)ethene (white solid 85% yield) was obtained by DCM evaporation.

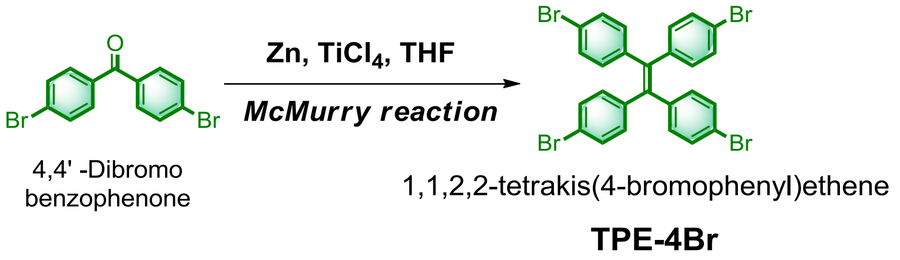

S1.2 Synthesis of TPA-TPA-MA.

Synthesis of TPA-TPA by Ullmann polymerization.[1] TPA-3I, (3.115 g, 5 mmol) and $Pd(OAc)_2$ (0.056 g, 0.15 mmol) was added to a solution of K_2CO_3 (2.073 g, 15 mmol), DPPP (0.206 g, 0.5 mmol) in dehydrated DMF (150 mL) under N_2 atmosphere, and the mixture was heated at 110 °C for 72 h to afford brown suspensions. After cooling to ambient temperature, concentrated HCl (10 mL) was added to the mixture. After filtration, the residue was washed with H_2O, MeOH, $CHCl_3$, THF, and acetone, extracted by Soxhlet with H_2O, methanol, $CHCl_3$, acetone, and THF for 1 day, respectively, and dried at 100 °C under vacuum for 24 h, to afford off yellow powder (2.1 g, 67% yield). TPA-TPA (1.8 g), Methyl acrylate (0.774 g, 9 mmol), $Pd(OAc)_2$ (0.1 g, 0.045 mmol), DPPP (0.37 g, 0.9 mmol) and Et_3N (2.24 g, 10 mmol) were added into 100 mL of dry DMF. [2] After stirred for 72 h at 100 °C under N_2 atmosphere. After cooling the reaction mixture to ambient temperature, the residue was isolated by filtration, washed with H_2O, MeOH, $CHCl_3$, THF, and acetone, and dried in vacuo to give was an bright yellow solid (0.9 g, 65% yield). **IR (KBr):** 3031, 1703, 1601, 1485, 1435, 1324, 1267, 1177, 1103, 820, 740, 690, 598, 529 cm^{-1}.

S1.3 Synthesis of TPA-PB-MA.

Synthesis of TPA-PB by Suzuki polymerization. [3] A dried 250 mL round-bottom flask was charged with TPA-3I (1.869 g, 3mmol), PB (0.747 g, 4.5 mmol), K_2CO_3 (1.242 g, 9 mmol) and $Pb(PPh_3)_4$ (0.3 mmol, 0.346 g) Then the mixture of THF (80 mL) and MeOH (80 mL) were added. And the reaction was refluxed at 120 °C for 48 h under N_2 atmosphere. The mixture was allowed to cool at ambient temperature and poured into water. The precipitate was collected by filtration, thoroughly washed with H_2O, MeOH, $CHCl_3$, THF, and acetone, extracted by Soxhlet with H_2O, methanol, $CHCl_3$, acetone, and THF for 1 day, respectively, and dried at 100 °C under vacuum for 24 h to afford white solid. (1.335g, 78% yield). TPA-PB (0.9 g) Methyl acrylate (0.517 g, 6 mmol), $Pd(OAc)_2$ (0.05 g, 0.025 mmol), DPPP (0.186 g, 0.45 mmol) and Et_3N (1.347 g, 6 mmol) were added into 80 mL of dry DMF respectively, using procedures similar to those for TPA-TPA-MA. Gray solid (0.6 g, 68% yield). **IR (KBr)**: 3026, 1599, 1477, 1326, 1282, 1183, 1116, 995, 954, 813, 743, 673, 592, 522 cm^{-1}.

S1.4 Synthesis of TPA-TFB-MA.

Synthesis of TPA-TFB by Suzuki polymerization. A dried 250 mL round-bottom flask was charged with Tris(4-iodophenyl)amine (1.246 g, 2 mmol), TFB (1.927 g, 3 mmol) K_2CO_3 (1.242 g, 9 mmol) and $Pb(PPh_3)_4$ (0.346 g, 0.3 mmol) Then the mixture of THF (80 mL) and MeOH (80 mL) were added. Using procedures similar to those for TPA-PB. Yellow-brown solid, yield 80%. TPA-TFB (0.89 g), Methyl acrylate (0.517 g, 6 mmol), $Pd(OAc)_2$ (0.05 g, 0.025 mmol), DPPP (0.186 g, 0.45 mmol) and Et_3N (1.347 g, 6 mmol) were added into 80 mL of dry DMF respectively, using procedures similar to those for TPA-TPA-MA. Khaki solid, yield 65%. **IR (KBr)**: 2930, 2851, 1605, 1509, 1463, 1311, 1280, 1181, 1108, 1012, 956, 888, 809, 735, 623, 538 cm^{-1}.

S1.5 Synthesis of TPA-TPE-MA.

Synthesis of TPA-TPE by Ullmann polymerization. Tris(4-iodophenyl)amine (0.958 g, 2 mmol), TPE(0.972g, 1.5mmol) $Pd(OAc)_2$ (0.067g, 0.3 mmol) K_2CO_3 (2.073 g, 15 mmol), DPPP (0.248 g, 0.6 mmol) and DMF (150 mL) respectively, using procedures similar to those for TPA-TPA. While solid, yield 70%. TPA-TPE (0.841g), Methyl acrylate (0.517 g, 6 mmol), $Pd(OAc)_2$ (0.05 g, 0.025 mmol), DPPP (0.186 g, 0.45 mmol) and Et_3N (1.347 g, 6 mmol) were added into 80 mL of dry DMF respectively, using procedures similar to those for TPA-TPA-MA. Yellow-brown solid, yield 68%. **IR (KBr)**: 3026, 2927, 2845, 1722, 1593, 1488, 1320, 1268, 1180, 1110, 999, 924, 819, 732, 697, 540 cm^{-1}.

S1.6 Synthesis of TPE-PB-MA.

Synthesis of TPE-PB by Suzuki Polymerization. TPE-4Br (1.3 g, 2 mmol), PB (0.664 g, 4 mmol), K_2CO_3 (1.242 g, 9 mmol), $Pb(PPh_3)_4$ (0.462 g,0.4 mmol) THF (80 mL) and MeOH (80 mL) respectively, using procedures similar to those for TPA-PB. Yellow-brown solid, yield 68%. TPE-PB (0.9 g) Methyl acrylate (0.517 g, 6 mmol), $Pd(OAc)_2$ (0.05 g, 0.025 mmol), DPPP (0.186 g, 0.45 mmol) and Et_3N (1.347 g, 6 mmol) were added into 80 mL of dry DMF respectively, using procedures similar to those for TPA-TPA-MA. Yellow solid, yield 70%. **IR (KBr)**: 3014, 1896, 1739, 1599, 1477, 1384, 1326, 1273, 1221, 1180, 1110, 1005, 930, 859, 801, 732, 679, 633, 586, 528 cm^{-1}.

S1.7 Synthesis of TPE-TFB-MA.

Synthesis of TPE-TFB by Suzuki Polymerization. TPE-4Br (0.777 g, 1.2mmol) TFB (1.29 g, 2 mmol) K_2CO_3 (1.111 g, 8 mmol) and $Pb(PPh_3)_4$ (0.23 g, 0.2 mmol) Then the mixture of THF (80 mL) and MeOH (80 mL) were added. Using procedures similar to those for TPA-PB. Yellow solid, yield 78%. TPE-TFB (0.89 g), Methyl acrylate (0.517 g, 6 mmol), $Pd(OAc)_2$ (0.05 g, 0.025 mmol), DPPP (0.186 g, 0.45 mmol) and Et_3N (1.347 g, 6 mmol) were added into 80 mL of dry DMF respectively, using procedures similar to those for TPA-TPA-MA. Green solid, yield 65%. **IR (KBr)**: 2915, 2851, 1599, 1512, 1454, 1355, 1244, 1197, 1017, 807, 726, 656, 595, 516 cm^{-1}.

S1.8 Fluorescence quantum yield (Φ_F) measurement.

Fluorescence quantum yield measurements were measured using a Fluoro Sens 9003 Fluorescence Spectrophotometer. The fluorescence quantum yields of conjugated polymers in THF at 25 ^oC were Calculated using integrated sphere. The fluorescence quantum yields were collected at the excitation wavelengths as399 and 325 nm (TPA-PB-MA, TPE-PB-MA, and TPE-TFB-MA were 399nm, TPA-TPA-MA, TPA-TPE-MA, TPA-TFB-MA were 325 nm). And each emission spectrum was collected from 500 to 750 nm with 0.5 nm step and 100 ms dwell time.

S1.9 Isosteric Heat of Adsorption (Q_{st}) Calculations.

The isosteric heat of adsorption (Q_{st}) was calculated using hydrogen adsorption isotherms measured at 273K and 298 K based on the integral format of Clausius-Clapeyron equation:[4]

$$\ln P_2 - \ln P_1 = \frac{Q_{st}}{R}\left(\frac{1}{T_1} - \frac{1}{T_2}\right)$$

where Qst is the heat of adsorption at a pressure P and temperature T; R is the universal gas constant (8.314 J K^{-1} mol^{-1}).

S1.10 Ideal adsorption solution theory (IAST) calculations. [5]

The IAST calculations were carried out for binary mixture containing 15/85 or 25/75 (v/v) CO_2 and N_2. Pure component gas adsorption isotherms were fitted using the nonlinear regression tool within the OriginPro8.5. The standard Langmuir model was used for N_2 adsorption isotherms fitting, dual-site Langmuir model was used for CO_2 adsorption isotherms fitting as it gives better simulations within the range of interest. The used equations are given below:

$$V_{abs} = q * a * p/(1 + a * p)$$

$$V_{abs} = \frac{q * a * p}{1 + a * p} + \frac{u * b * p}{1 + b * p}$$

where p is the pressure given in mmHg, V_{abs} is the adsorbed amount of gas in cm^3/g STP, a and b are Langmuir-type affinity constants, q and u represent the Langmuir-type adsorption capacity. The pure-component isotherm fitting parameters obtained above were then applied for IAST binary-gas adsorption selectivity (S) calculations based on the equation given below:

$$S = \frac{q_{CO_2}/q_{N_2}}{p_{CO_2}/p_{N_2}}$$

where q represents the amount adsorbed from the binary gas phase, and p represents gas phase composition (i.e. 15/85 and 25/75).

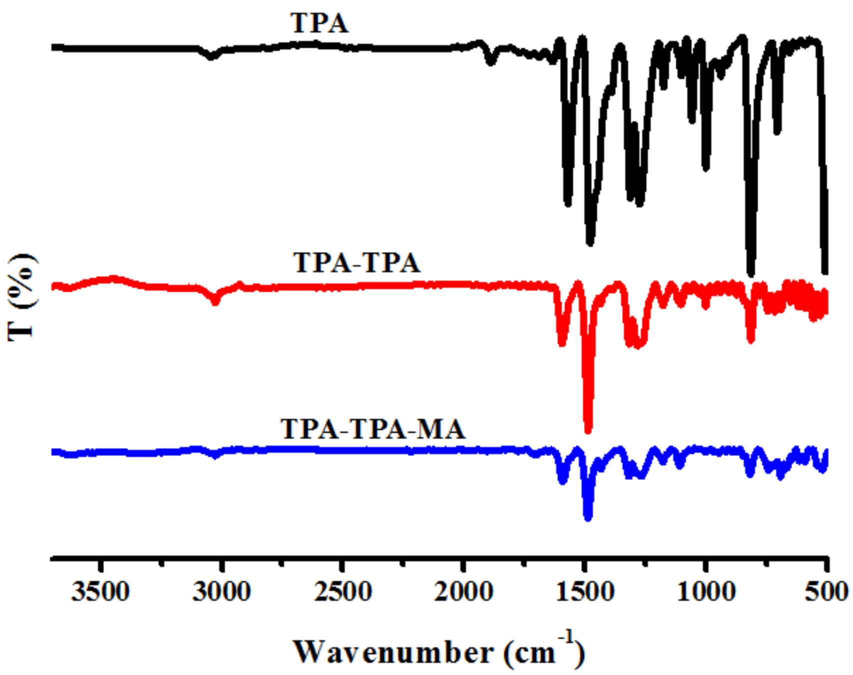

Fig. S1 FT-IR spectra of TPA, TPA-TPA and TPA-TPA-MA.

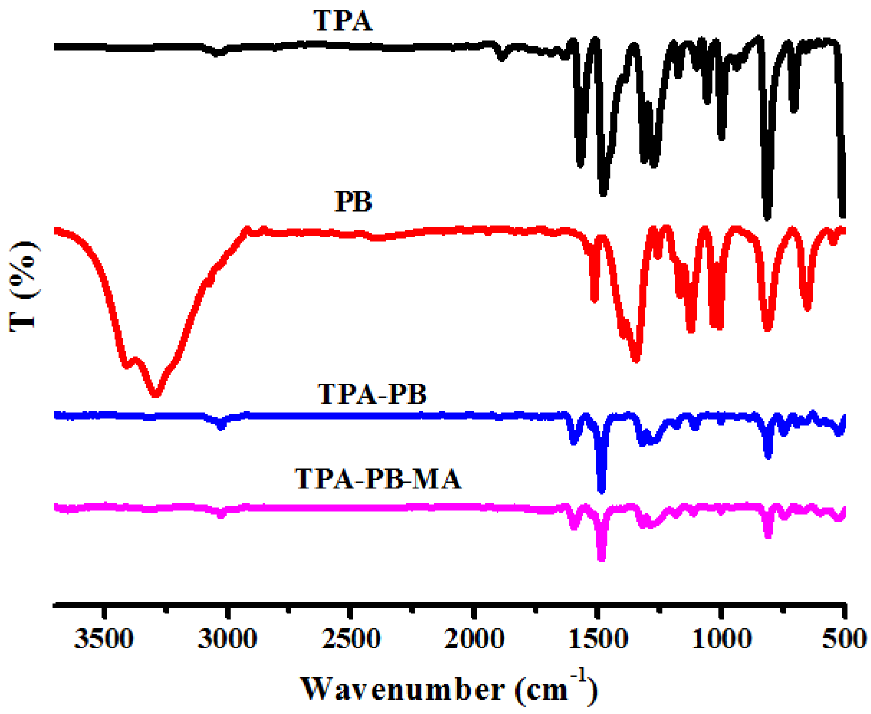

Fig. S2 FT-IR spectra of TPA, PB, TPA-PB and TPA-PB-MA.

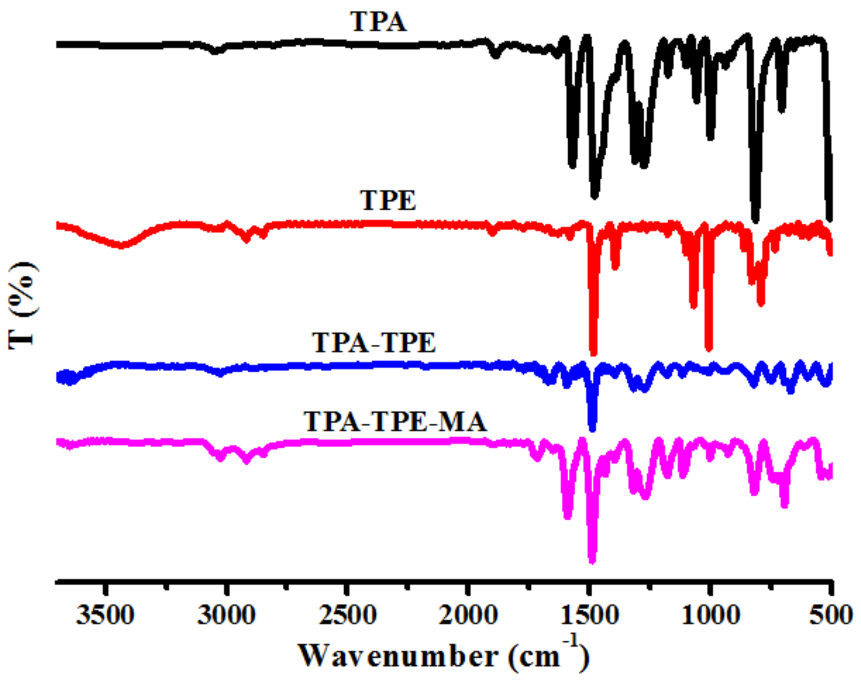

Fig. S3 FT-IR spectra of TPA, TFB, TPA-TFB and TPA-TFB-MA.

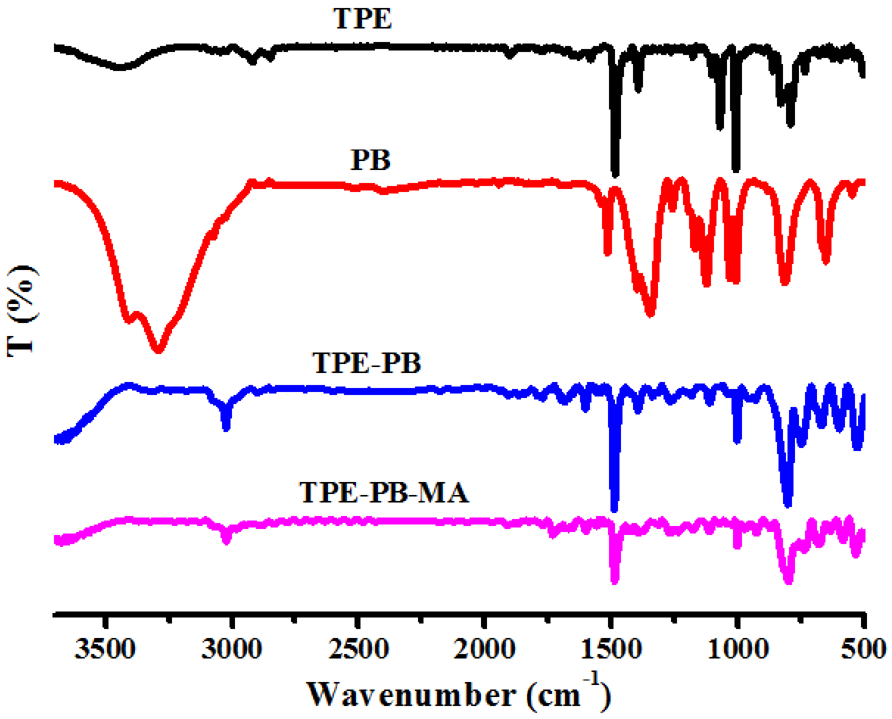

Fig. S4 FT-IR spectra of TPE, PB, TPE-PB and TPE-PB-MA.

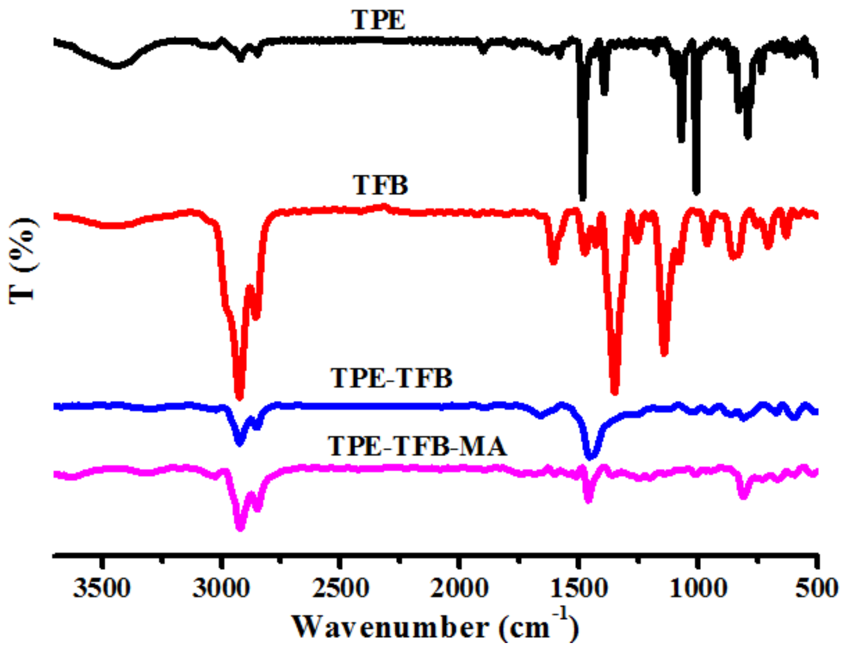

Fig. S5 FT-IR spectra of TPE, TFB, TPE-TFB and TPE-TFB-MA.

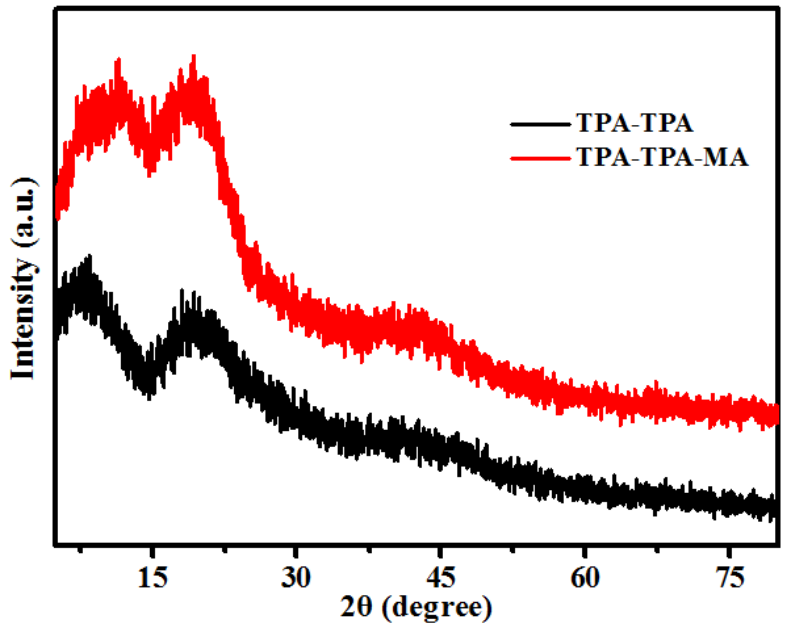

Fig. S6 X-ray diffraction profiles of TPA-TPA and TPA-TPA-MA.

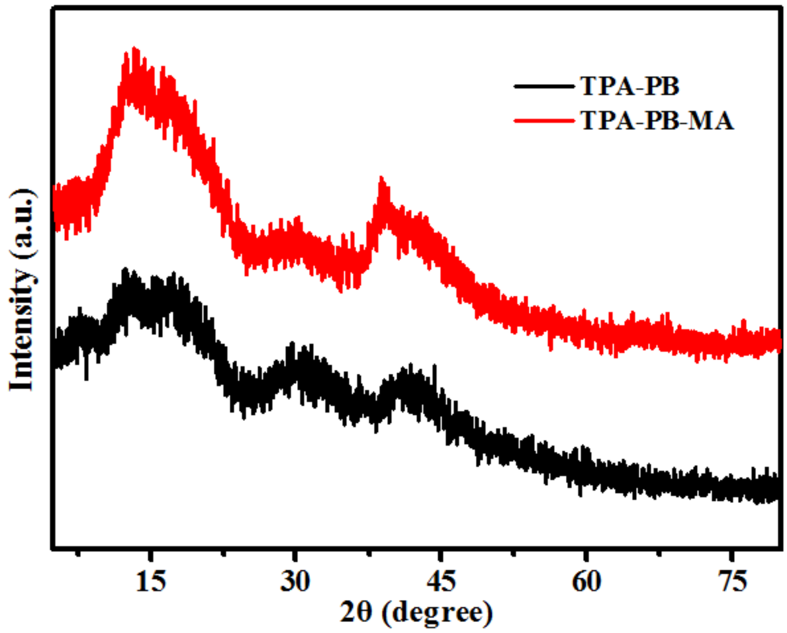

Fig. S7 X-ray diffraction profiles of TPA-PB and TPA-PB-MA.

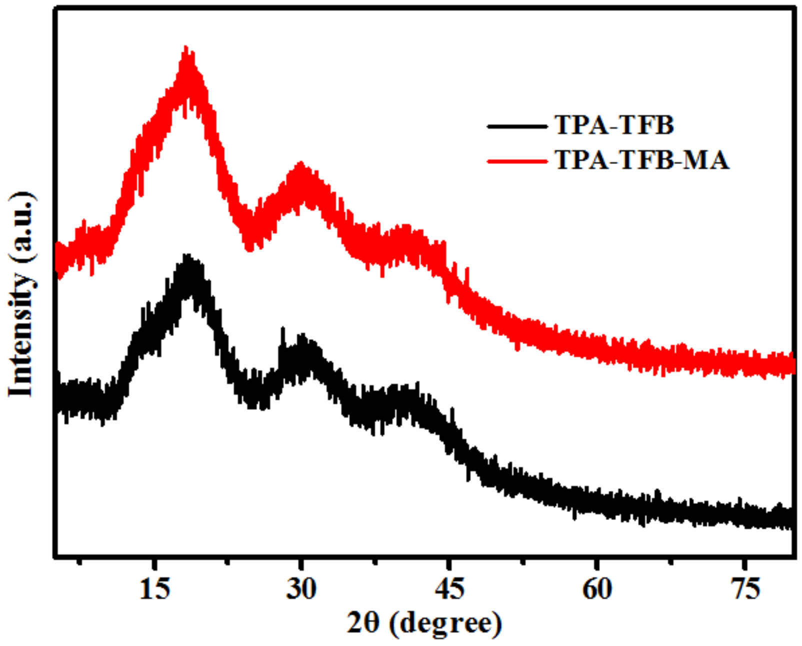

Fig. S8 X-ray diffraction profiles of TPA-TFB and TPA-TFB-MA.

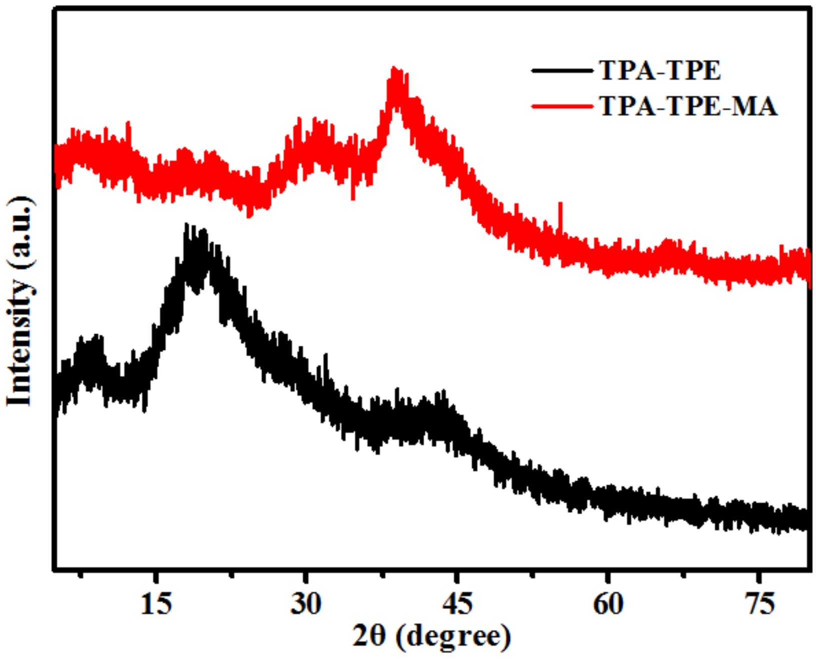

Fig. S9 X-ray diffraction profiles of TPA-TPE and TPA-TPE-MA.

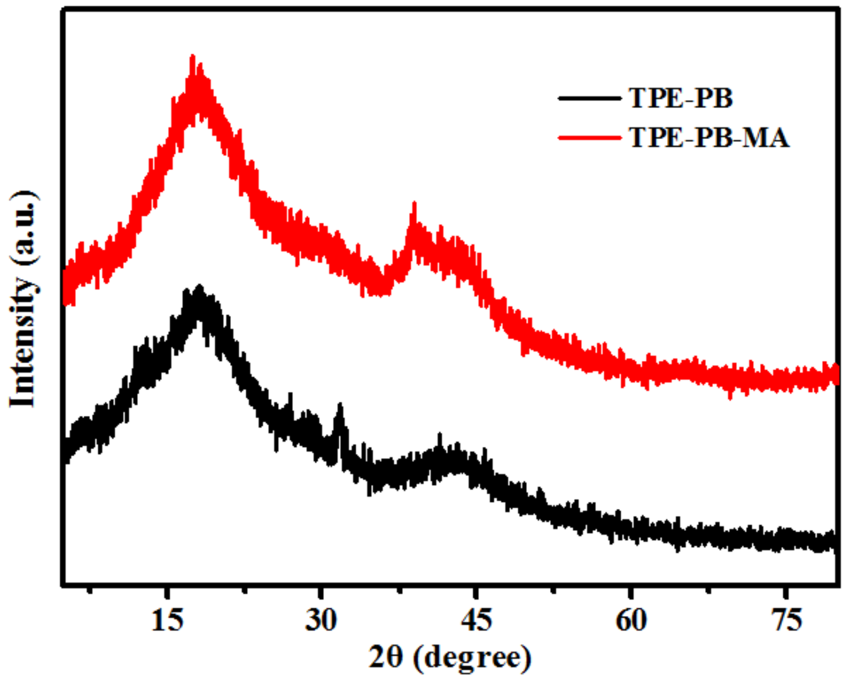

Fig. S10 X-ray diffraction profiles of TPE-PB and TPE-PB-MA.

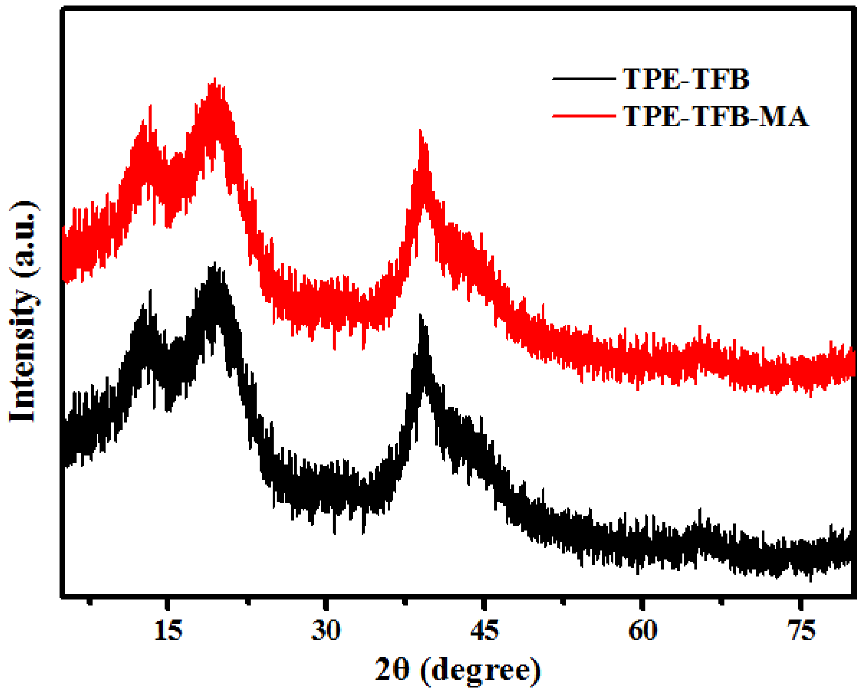

Fig. S11 X-ray diffraction profiles of TPE-TFB and TPE-TFB-MA.

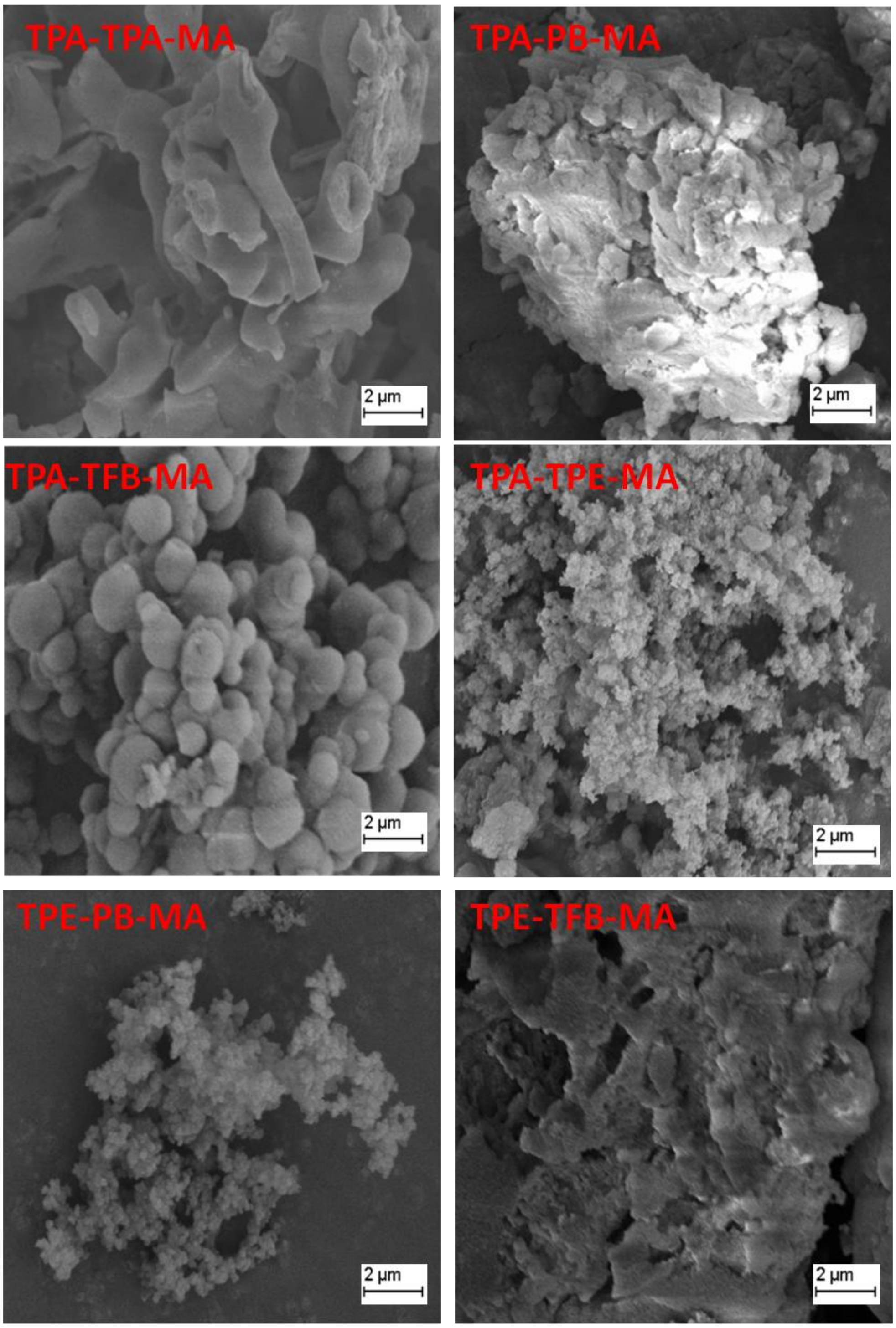

Fig. S12 FE-SEM images of conjugated polymers.

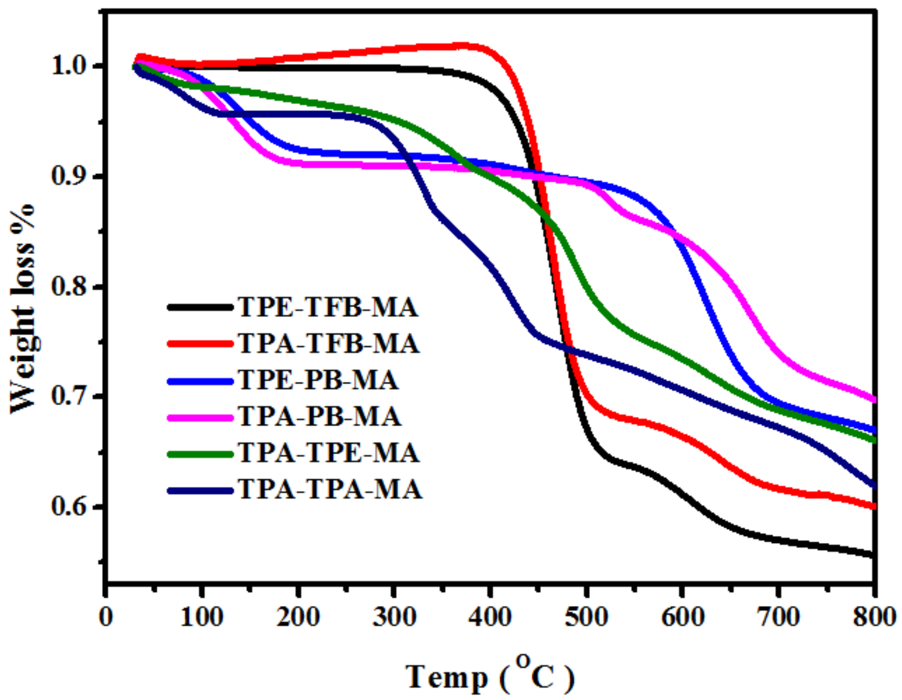

Fig. S13 Thermal gravimetric analysis of conjugated polymers.

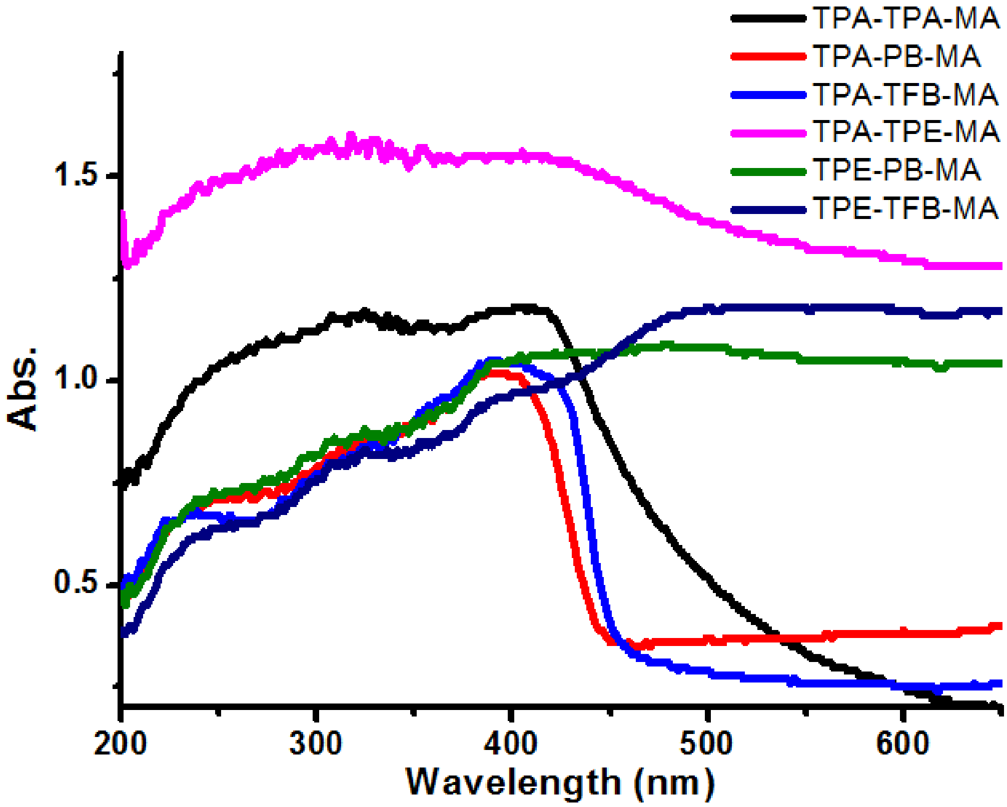

Fig. S14 UV-Vis absorption spectra of conjugated polymers in solid state.

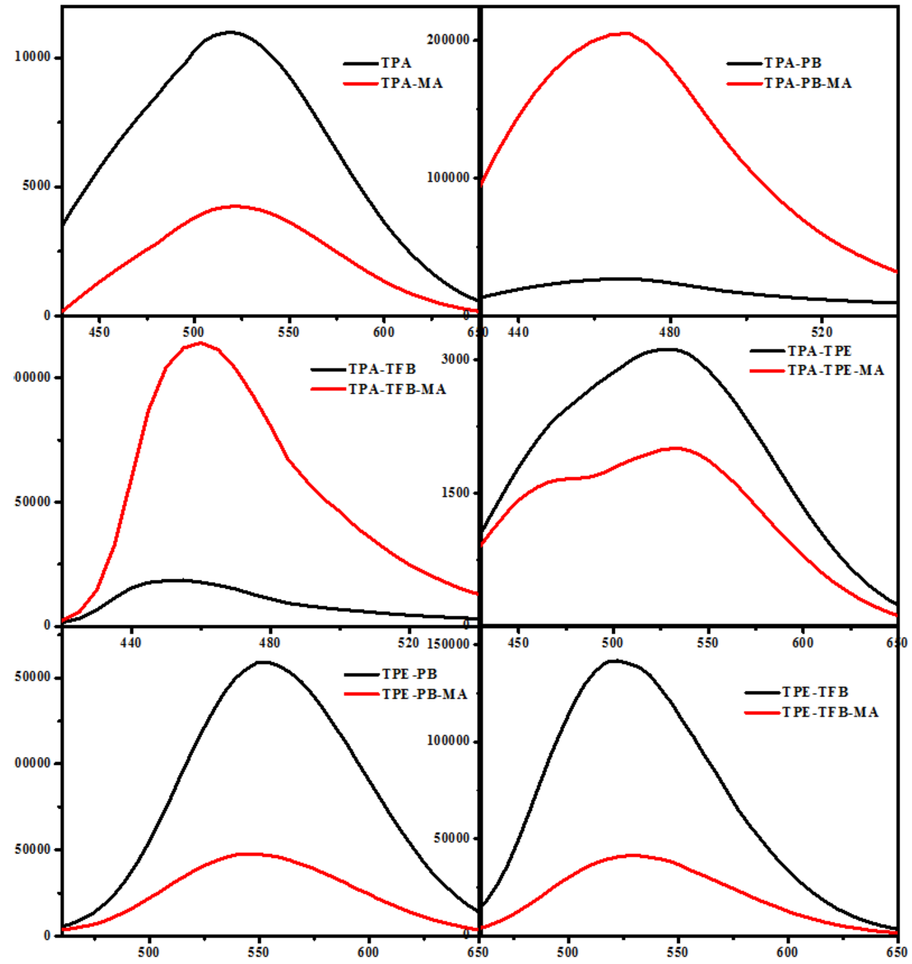

Fig. S15 Fluorescence spectra of conjugated polymers in solid state. (The excitation wavelength of TPA-TPA-MA, TPA-TPE-MA and TPA-TFB-MA were 325 nm; the excitation wavelength of TPA-PB-MA, TPE-PB-MA and TPE-TFB-MA were 399 nm.)

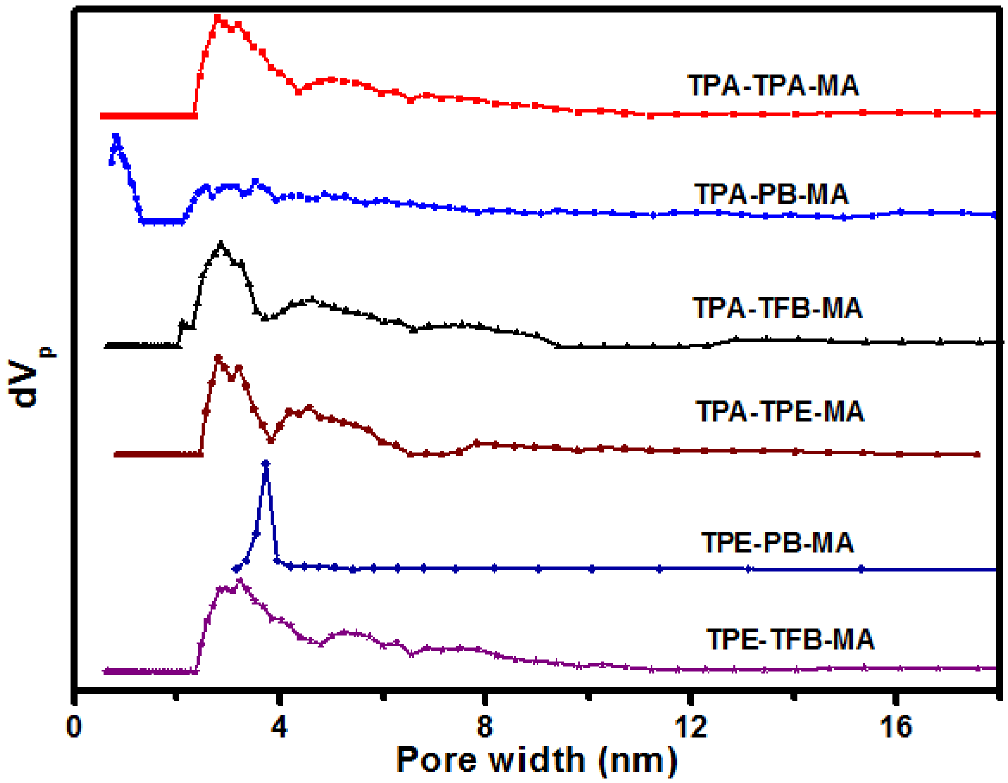

Fig. S16 Pore size distribution curves of polymers via NLDFT calculation.

Table S1 Porosity parameter of conjugated polymers.

	$S_{BET}(m^3/g)$	Pore volume (cm^3/g)	Pore size (nm)
TPA-TPA-MA	70.973	0.077	3.169
TPA-PB-MA	686.230	0.716	0.823
TPA-TFB-MA	41.694	0.055	2.269
TPA-TPE-MA	76.669	0.068	2.769
TPE-PB-MA	191.588	0.278	5.888
TPE-TFB-MA	36.319	0.043	4.887

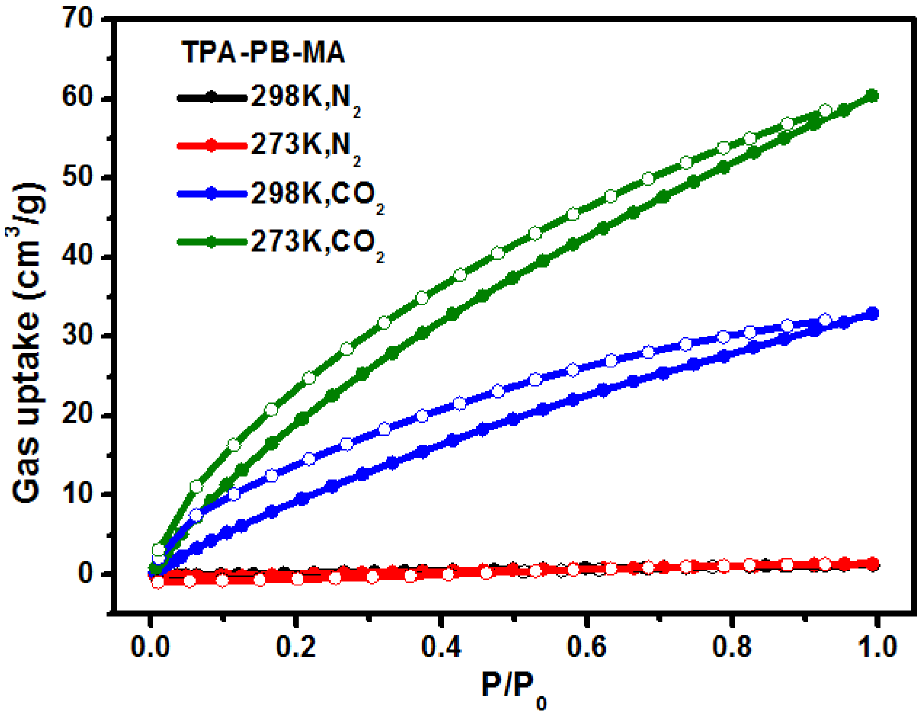

Fig. S17 Adsorption and desorption isotherm of CO_2 and N_2 of TPA-PB-MA at 298 and 273 K.

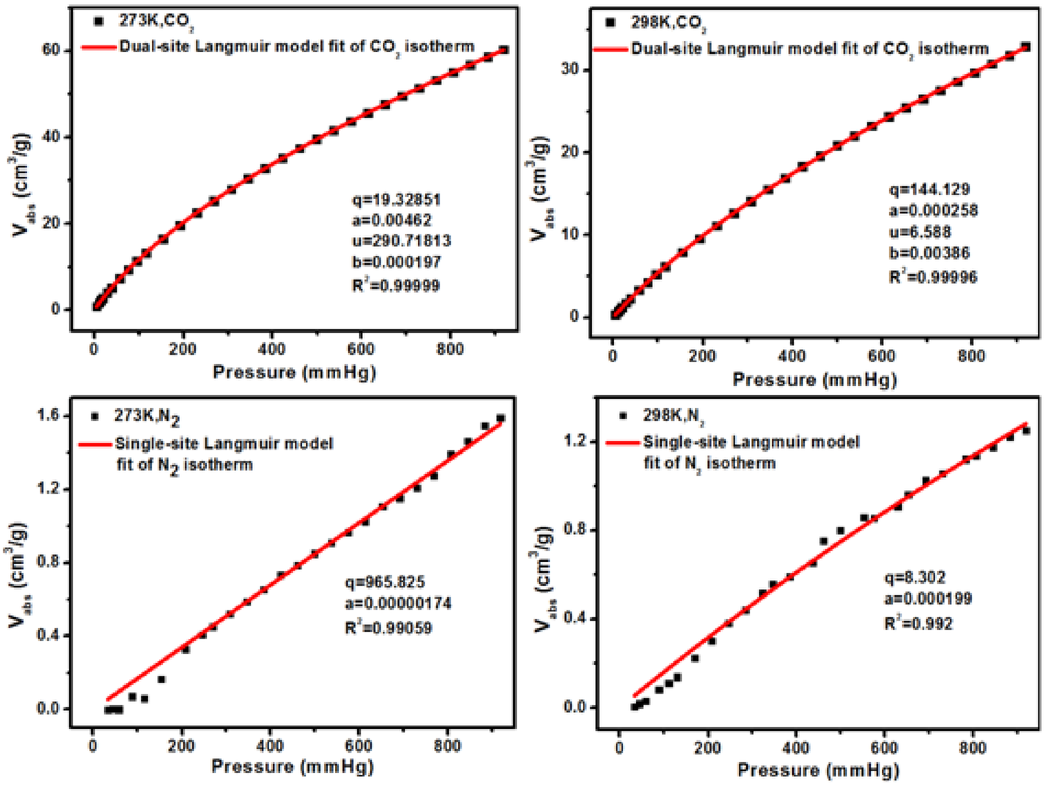

Fig. S18 Single-site model and dual-site Langmuir model fittings of N_2 and CO_2 isotherms of TPA-PB-MA.

Scheme S1. Structures of the different tested nitroarenes.

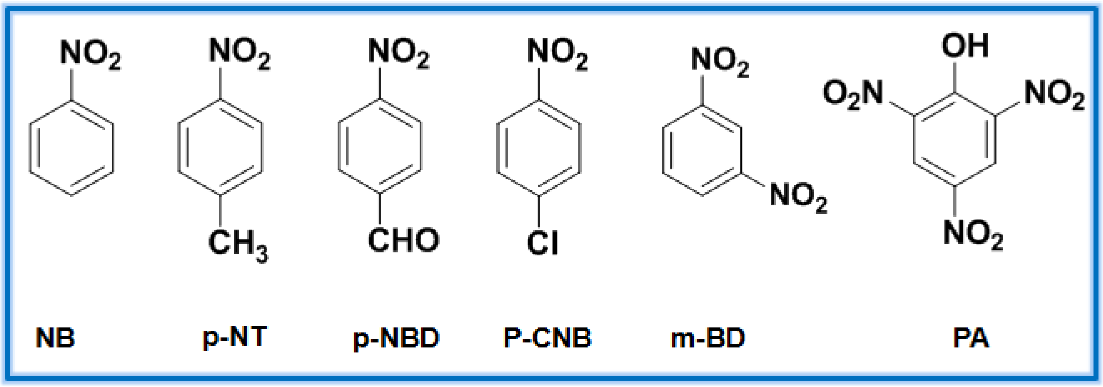

Table S2. The Ksv constants of conjugated polymers to the analytes.

Analyte	TPA-TPA-MA	TPA-PB-MA	TPA-TFB-MA	TPA-TPE-MA	TPE-PB-MA	TPE-TFB-MA
PA	1544	39176	11290	18954	2872	431
m-BD	4782	6242	1216	2036	284	1560
NB	7650	3978	2676	1250	746	5280
p-NT	6688	3130	4182	2384	352	841
P-CNB	2928	3220	4744	442	656	6062
P-NBD	7726	3655	6122	476	2321	1518

Table S3. Summary of K_{SV} of polymers.

Polymers	$K_{SV}/M^{-1}(10^{-4})$	Ref.	Polymers	$K_{SV}/M^{-1}(10^{-4})$	Ref.
TPA-TPA-MA	0.15	Our work	COP-3	1.45	8
TPA-PB-MA	4.00		COP-4	0.39	
TPA-TFB-MA	1.13		CMP-LS1	5.05	9
TPA-TPE-MA	1.89		CMP-LS2	3.70	
TPE-PB-MA	0.28		LMOP-15	2.60	10
TPE-TFB-MA	0.04		DTF	2.08	11
PU-PF	2.91	6	Zn-TCPP	3.59	12
PU-PFTPE	1.15		TPE-CMP	1.19	13
PU-PTTPE	0.83		2	4.75	14
BTB	0.71	7	3	1.05	

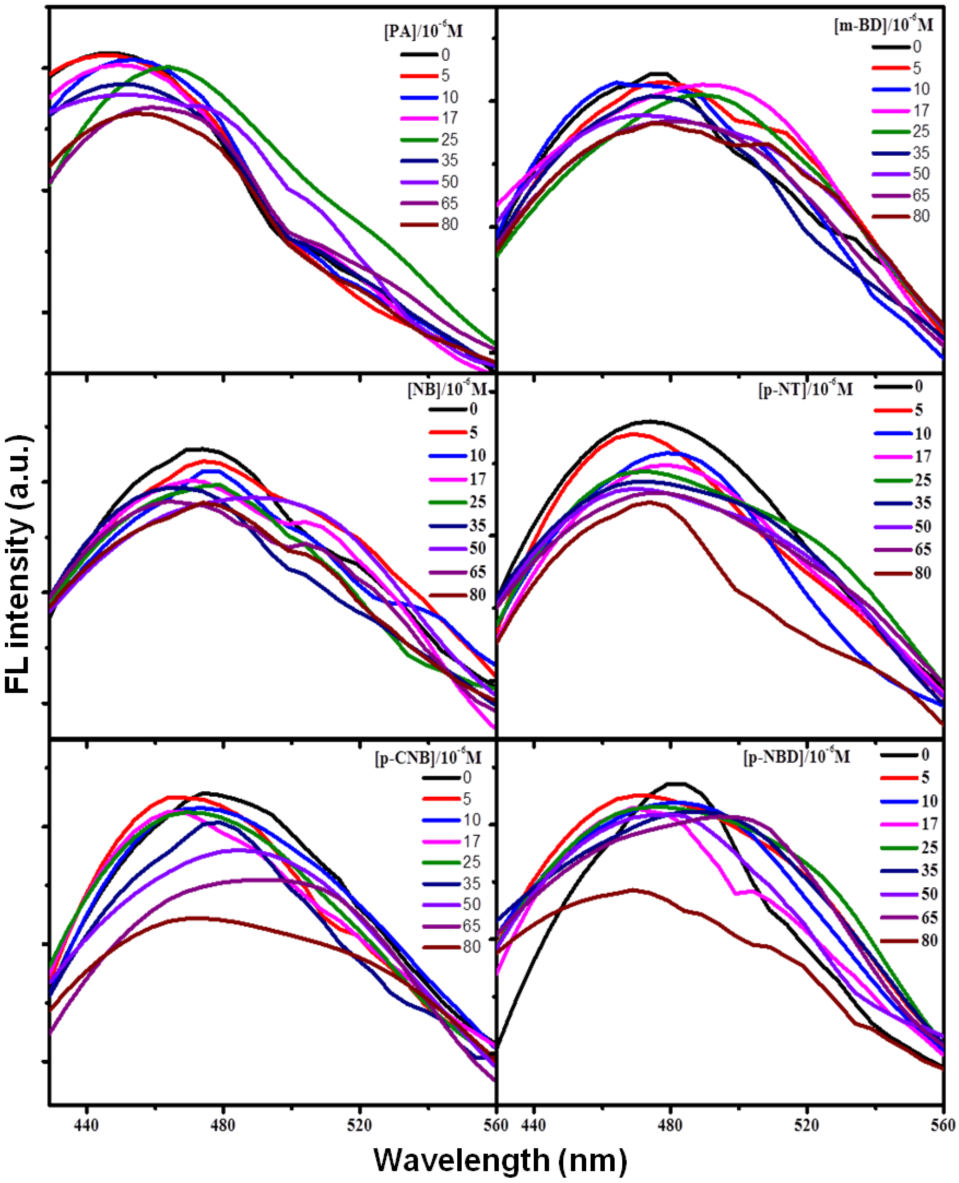

Fig. S19 Fluorescence spectra of TPA-TPA-MA to the analytes in THF.

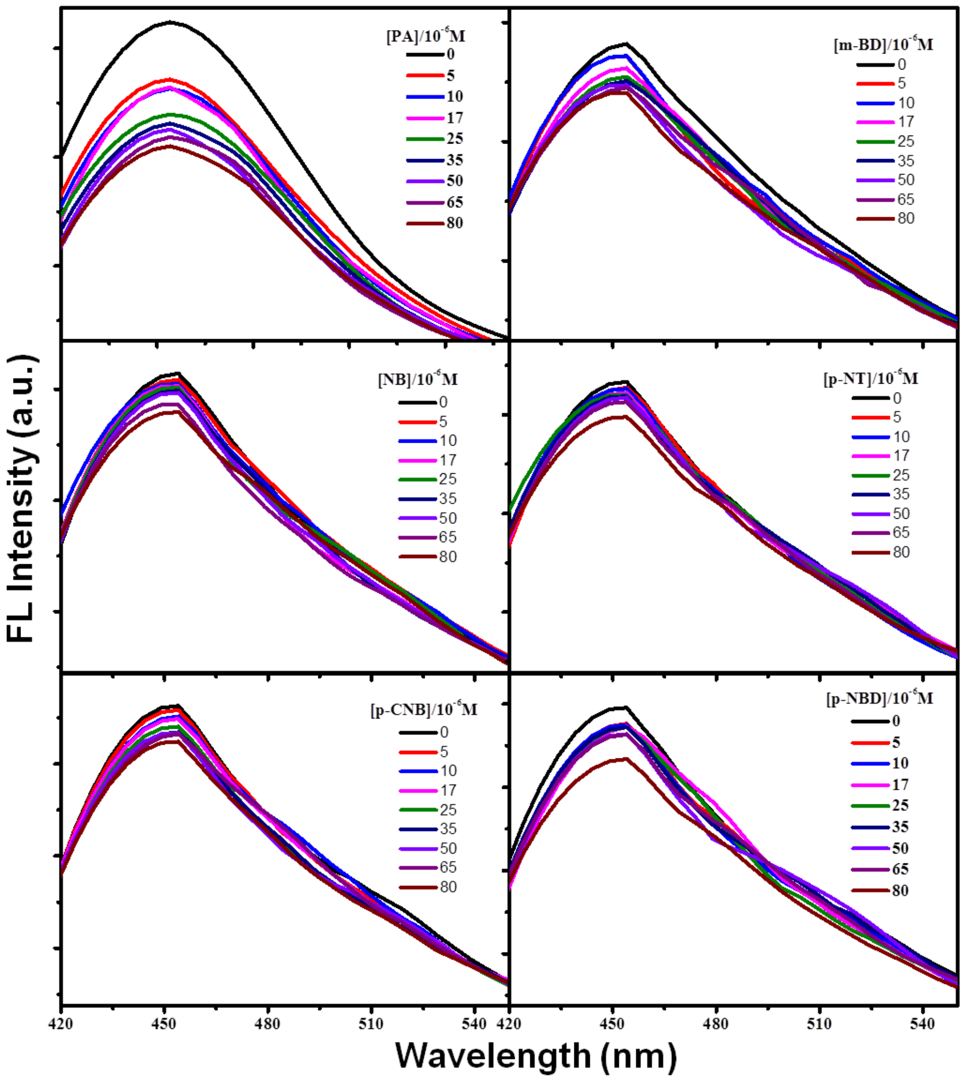

Fig. S20 Fluorescence spectra of TPA-PB-MA to the analytes in THF.

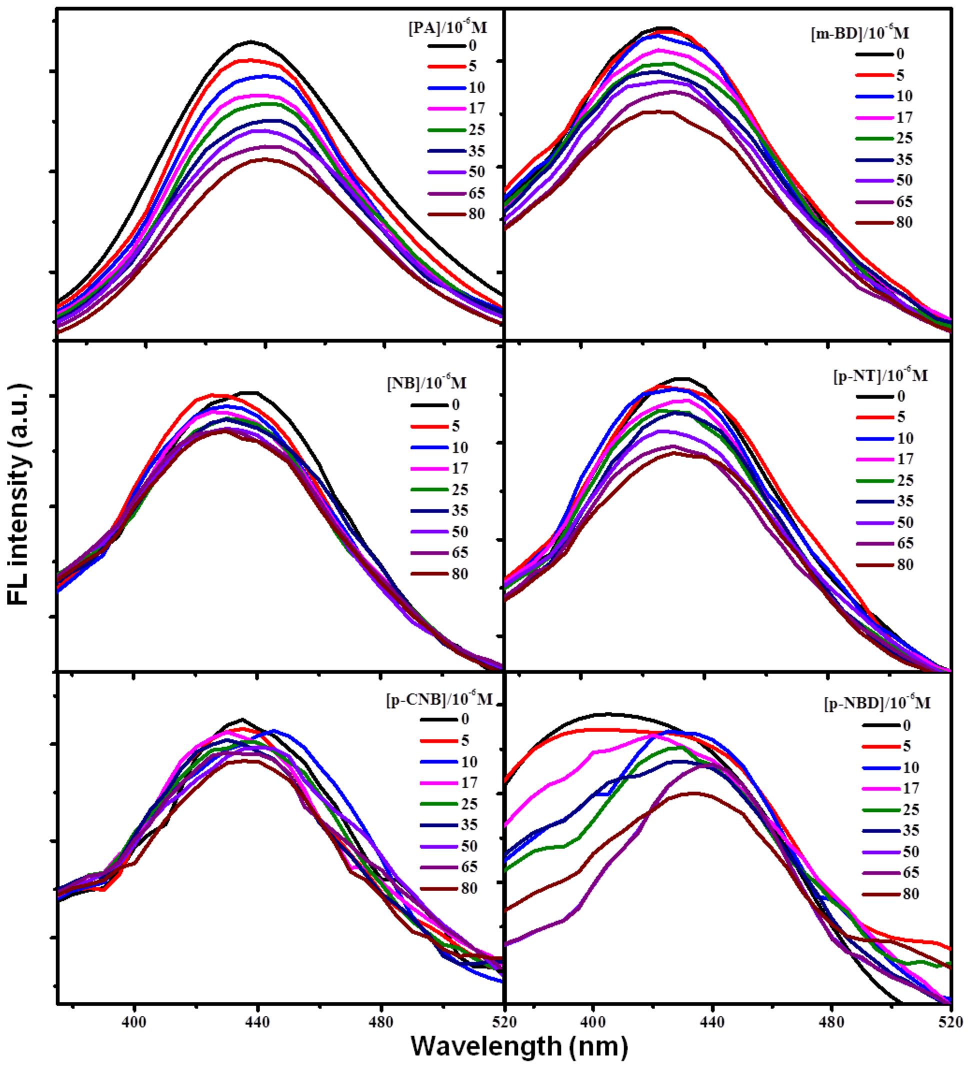

Fig. S21 Fluorescence spectra of TPA-TFB-MA to the analytes in THF.

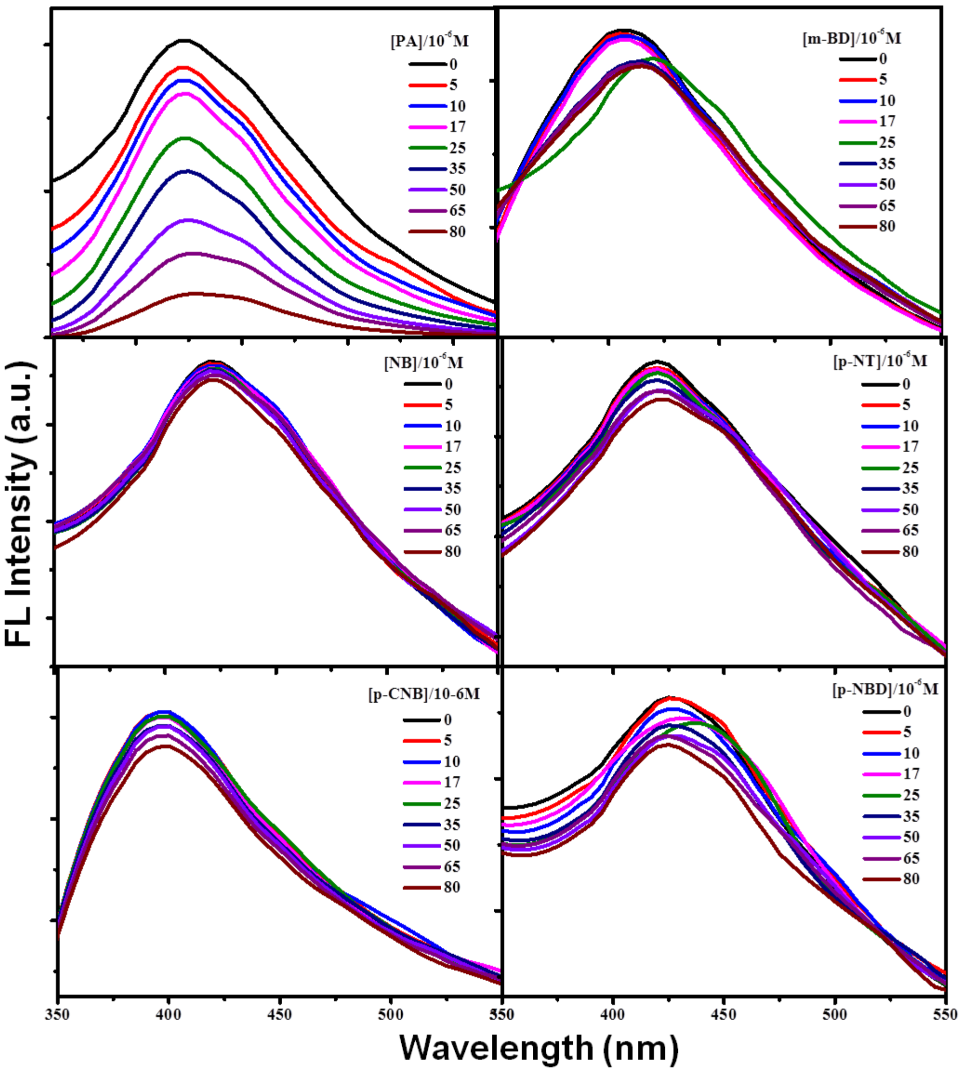

Fig. S22 Fluorescence spectra of TPA-TPE-MA to the analytes in THF.

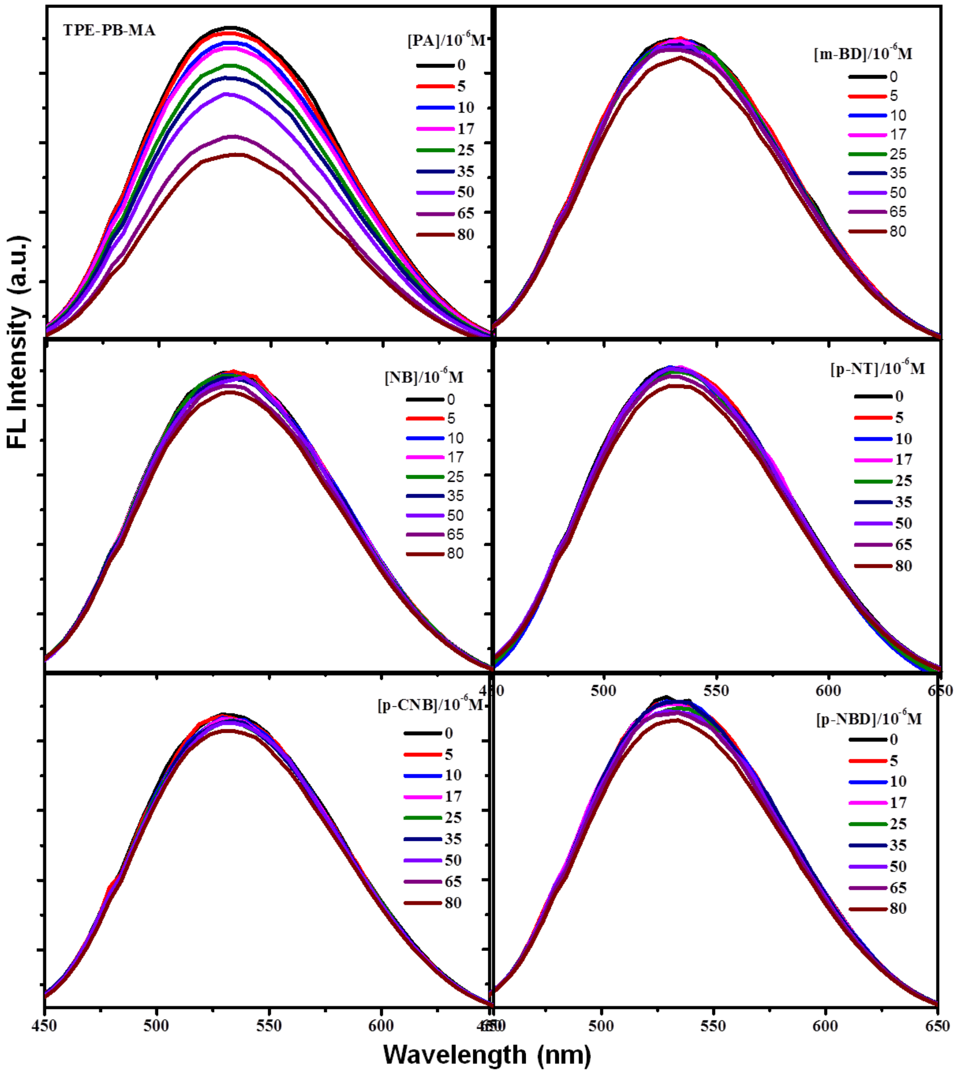

Fig. S23 Fluorescence spectra of TPE-PB-MA to the analytes in THF.

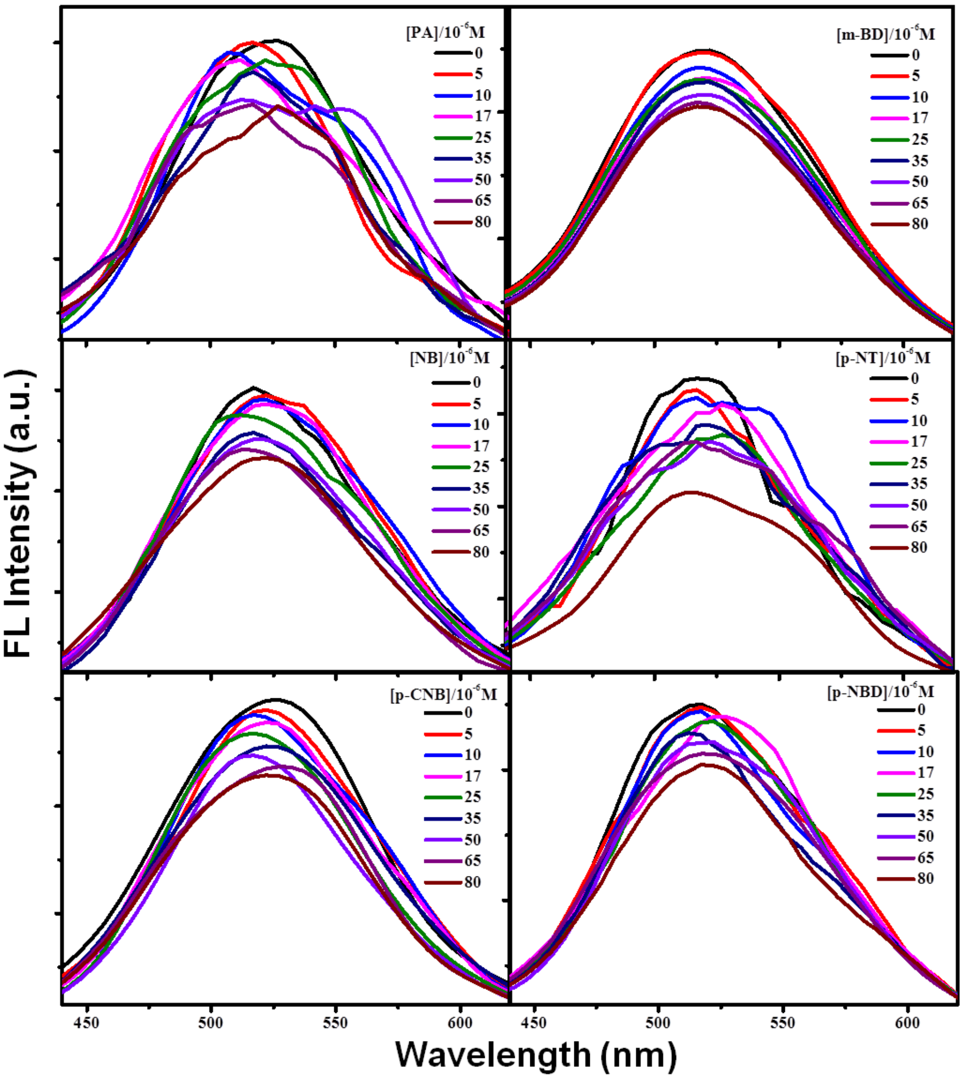

Fig. S24 Fluorescence spectra of TPE-TFB-MA to the analytes in THF.

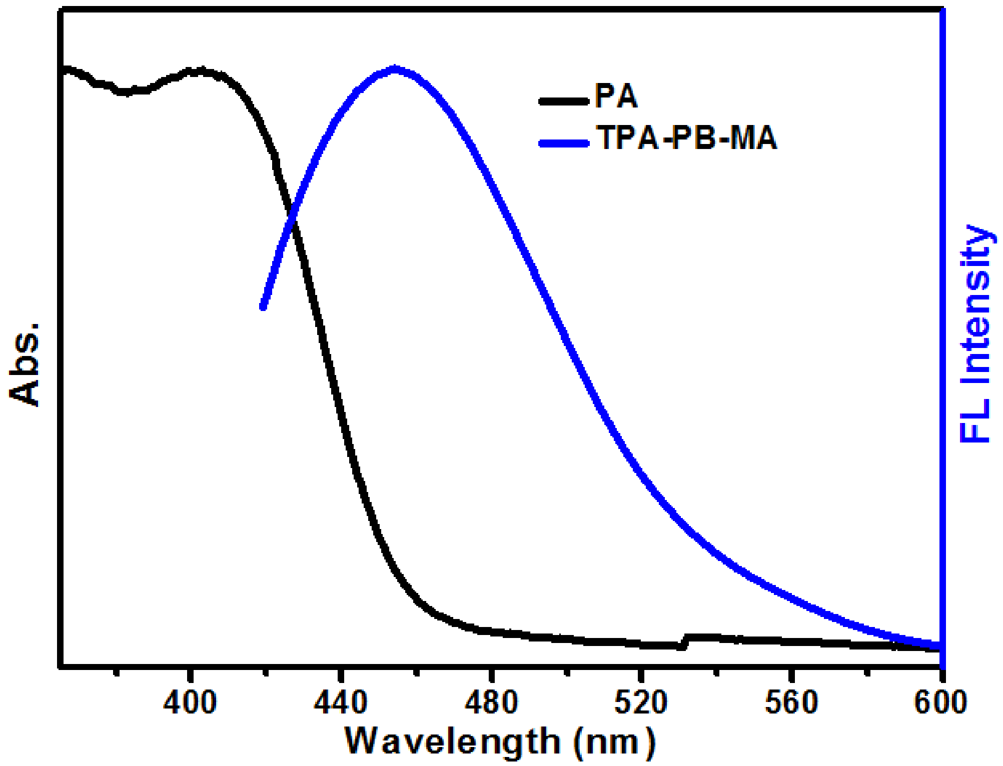

Fig. S25 UV-Vis absorption spectra of PA and fluorescence spectra of TPA-PB-MA dispersed in THF (λ_{ex} = 399 nm).

References

(1) G. Zhou, M. Baumgarten and K. Müllen, *J. Am. Chem. Soc.* 2007, **129**, 12211 – 12221.

(2) S. T. Pasco, P. M. Lahti and F. E. Karasz, *Macromolecules*, 1999, **32**, 6933 – 6937.

(3) J. J. Davis, C. B. Bagshaw, K. L. Busuttil and B. Hanyu, *J. Am. Chem. Soc.*, 2006, **128,** 14135 –14141.

(4) Y. Liao, Z. Cheng, W. Zuo, A. Thomas and C. F. J. Faul, *ACS Appl. Mater. Interfaces.* 2017, **9,** 38390 -38400.

(5) Y. Yuan, H. Huang, L. Chen and Y. Chen, *Macromolecules*, 2017, **50**, 4993 - 5003.

(6) Z. Chu, Z. Fan, X. Zhang, X. Tan, D. Li, G. Chen and Q. Zhao, *Sensors* 2018, **18**, 1565.

(7) N. Shi, Y. Zhang, D. Xu, C. Song, X. Jin, D. Liu, L. Xie and W. Huang, *New J. Chem.,* 2015, **39**, 9275-9280.

(8) Z. Xiang and D. Cao, *Macromol. Rapid Commun.* 2012*,* **33***,* 1184-1190.

(9) S. Wang, Y. Liu, Y. Yu, J. Du, Y. Cui, X. Song and Z. Liang, *New J. Chem.* 2018, **42***,* 9482-9487.

(10) Y. Cui, Y. Liu, J. Liu, J. Du, Y. Yu, S. Wang, Z. Liang and J. Yu, Polym. Chem., 2017, **8**, 4842-4848.

(11) T.-M. Geng, S.-N. Ye, Y. Wang, H. Zhu, X. Wang and X. Liu, Talanta, 2017, 165, 282-288.

(12) Y. Jiang, L. Sun, J. Du, Y. Liu, H. Shi, Z. Liang and J. Li, Crystal Growth & Design, 2017, **17**, 2090-2096.

(13) H. Namgung, J. J. Lee, Y. J. Gwon. and T. S. Lee, *RSC Adv.*, 2018, **8**, 34291-34296.

(14) N. Wang, J.-C. Yang, L.-D. Chen, J. Li, Y. An, C.-W. Lv and Y.-Q. Tian, *New J. Chem.*, 2017, **41**, 2786-2792.
